# Applications of CRISPR/Cas genome editing in economically important fruit crops: recent advances and future directions

**DOI:** 10.1186/s43897-023-00049-0

**Published:** 2023-01-28

**Authors:** Zhimin Ma, Lijing Ma, Junhui Zhou

**Affiliations:** Peking University Institute of Advanced Agricultural Sciences, Weifang, 261000 Shandong China

**Keywords:** CRISPR/Cas, Plant development, Plant immunity, Genome editing, Fruit crops

## Abstract

Fruit crops, consist of climacteric and non-climacteric fruits, are the major sources of nutrients and fiber for human diet. Since 2013, CRISPR/Cas (Clustered Regularly Interspersed Short Palindromic Repeats and CRISPR-Associated Protein) genome editing system has been widely employed in different plants, leading to unprecedented progress in the genetic improvement of many agronomically important fruit crops. Here, we summarize latest advancements in CRISPR/Cas genome editing of fruit crops, including efforts to decipher the mechanisms behind plant development and plant immunity, We also highlight the potential challenges and improvements in the application of genome editing tools to fruit crops, including optimizing the expression of CRISPR/Cas cassette, improving the delivery efficiency of CRISPR/Cas reagents, increasing the specificity of genome editing, and optimizing the transformation and regeneration system. In addition, we propose the perspectives on the application of genome editing in crop breeding especially in fruit crops and highlight the potential challenges. It is worth noting that efforts to manipulate fruit crops with genome editing systems are urgently needed for fruit crops breeding and demonstration.

**Graphical Abstract:**

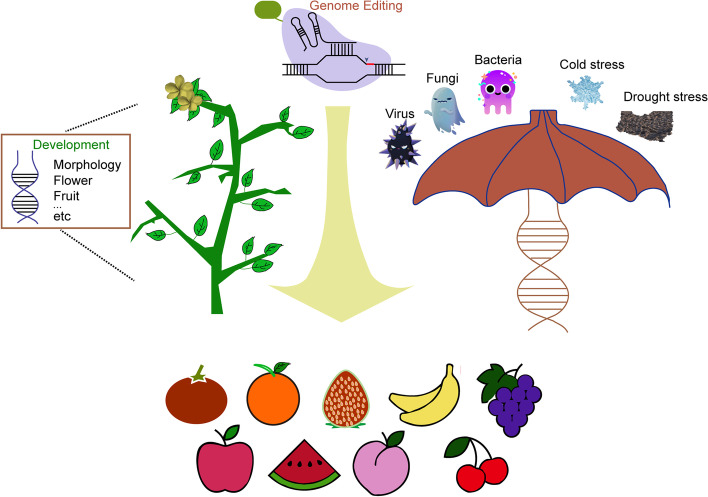

## Introduction

Fruits and vegetables greatly contribute to agricultural production, as they are major nutrient supplies for human and contain critical components for a healthy diet. Fruit crops have been cultivated for centuries. However, their production is hampered by many biological and environmental factors, including the relatively long life-cycle, the highly heterozygous genomes, the unfavorable climate and the limited availability of arable land (Dalla Costa et al. 2017; Leisner 2020). Therefore, improving fruit crop productivity and sustainability through scientific advancements and technological innovations is required urgently.

Over the long history of crop domestication, four major plant breeding techniques have been developed and exploited: 1) conventional breeding by crossing and selection; 2) mutation-based plant breeding; 3) transgene-based plant breeding; and 4) the genome editing-based plant breeding (Hickey et al. 2019). The traditional breeding by hybridization and mutation-based breeding usually take decades and are labor-intensive (Chen et al. 2019). The transgenic plant breeding developed rapidly since the last century and emerged as one of the most promising ways to accumulate several elite traits in one variety, even though this technology aroused a lot of controversies soon after its birth, mainly due to the safety concerns and ethical issues (Prado et al. 2014).

**Fig. 1:**
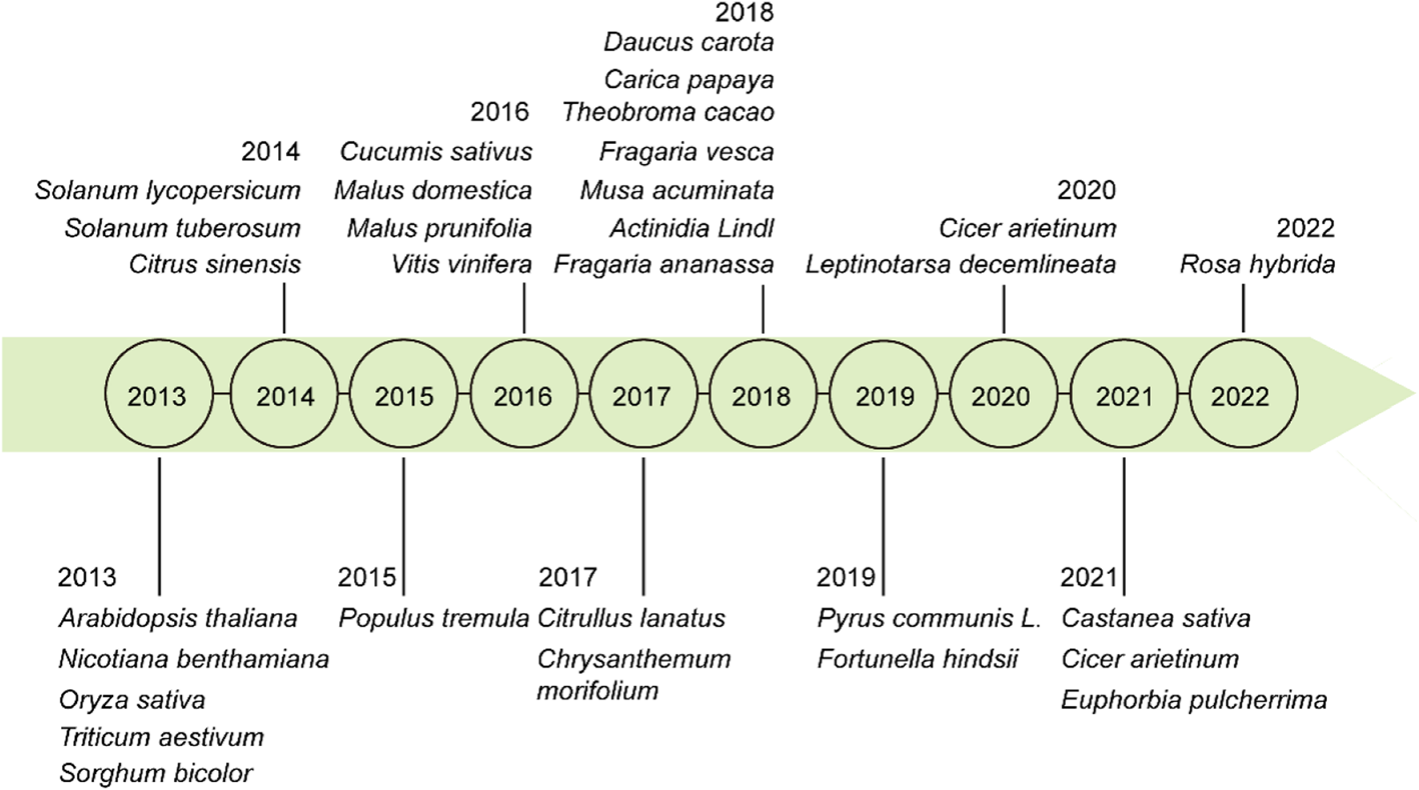
Timeline of CRISPR/Cas9 system was first applied in different plant species

Genome editing has been developed to obtain desired plant traits, as it could generate precise genome modification. Many systems have been developed to achieve genome editing in plants, including zinc-finger nucleases (ZFNs), transcription activator-like effector nucleases (TALENs) and the clustered regularly interspersed short palindromic repeats (CRISPR)/Cas system (Voytas and Gao 2014). CRISPR/Cas system has become one of the most widely used systems due to its low-cost, easy-to adapt and high specificity during genetic manipulation (Yin et al. 2017). This technology has been successfully applied in many cereals and economically important crops such as rice (*Oryza sativa*) (Shan et al. 2013), wheat (*Triticum aestivum*) (Shan et al. 2013), maize (*Zea mays*), potato (*Solanum tuberosum*) (Wang et al. 2015a), cassava (*Manihot esculenta*) (Odipio et al. 2017), chrysanthemums (*Chrysanthemum morifolium*) (Kishi-Kaboshi et al. 2017), European chestnut (*Castanea sativa*) (Pavese et al. 2021), Kabuli chickpea (*Cicer arietinum*) (Badhan et al. 2021), poinsettias (*Euphorbia pulcherrima*) (Nitarska et al. 2021) and rose (*Rosa hybrida*) (Wang et al. 2022a) (Fig. 1). Here we mainly describe status of CRISPR/Cas based genome editing in various fruit crops, emphasizing on plant development and disease resistance, especially plant architecture, fruit development, fruit ripening and quality, biotic stresses, and abiotic stresses in climacteric and non-climacteric fruits. We propose the future directions, especially the application of genome editing to promote plant breeding and overcome obstacles in fruit crops production.

## Overview of CRISPR/Cas genome editing

**Fig. 2:**
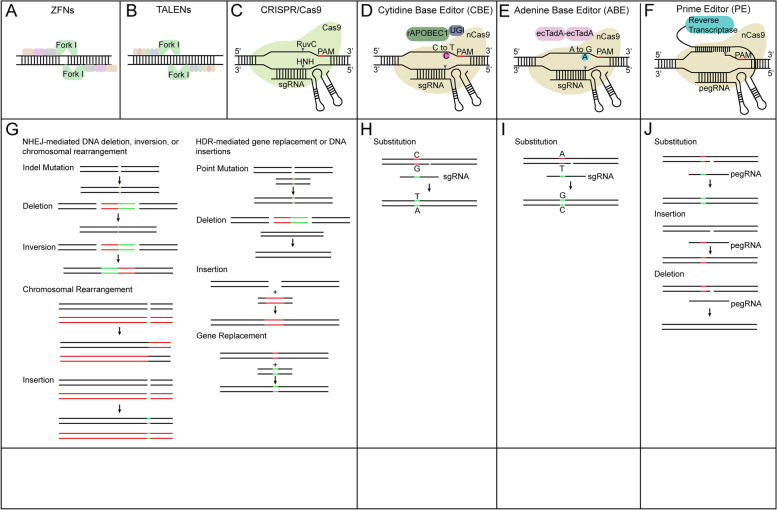
Schematics of genome editing technology systems and genome modifications generated by different systems. **A** ZFNs technology contains an array of engineered zinc finger proteins fused to catalytic domain of the FokI endonuclease. **B** TALENs have arrays of the TAL effector fused with FokI. **C** CRISPR/Cas9 system which is composed of Cas9 protein and sgRNA. (**D**) The cytidine base editor (CBE). Green circle represents cytidine deaminase rAPOBEC1. Purple circle represents uracil glycosylase inhibitor (UGI). **E** The adenine base editor (ABE). Light purple circle represents adenine deaminase TadA. **F** Prime editing technology. The prime editor (PE) is made up of a fusion protein of nCas9 (H840A) with reverse transcriptase and a prime editing guide RNA (pegRNA). **G** ZFNs, TALENs and CRISPR/Cas9 deliver double strand breaks (DSBs). DNA repair pathway includes the DNA non-homologous end joining (NHEJ) repair pathway and homology directed repair (HDR) pathway. DNA repair pathway produce different forms of genome modifications. **H** CBE generates base substitution of C•G to A•T without DSBs. **I** ABE make base substitution of A•T to G•C without DSBs. **J** PE generate precise genome modification of DNA substitution, insertion and deletion

Classical genome editing is accomplished via the DNA repair pathway after DNA double strand breaks (DSBs). Targeted DSBs are caused by sequence-specific nucleases (SSNs) enzymes which recognize and break DNA strand specifically (Voytas and Gao 2014). Four different SSNs have been adopted to introduce DSBs, including meganucleases, zinc finger nucleases (ZFNs) (Fig. 2A), transcription activator-like effector nucleases (TALENs) (Fig. 2B), and CRISPR/Cas reagents (Fig. 2C). When SSNs recognize and introduce DSBs, they are repaired by endogenous DNA repair pathways including non-homologous end joining (NHEJ) and homology-directed repair (HDR). The NHEJ repair pathway does not require a homologous repair template and usually introduces small insertions, deletions, or substitutions, eventually leading to genome modification and loss of gene function (Chen et al. 2019) (Fig. 2G). On the contrary, the HDR repair pathway requires a homologous DNA template to introduce insertions, mutations or replacements of DNA fragments (Gao 2021) (Fig. 2G). The application of meganucleases, ZFNs and TALENs is limited mainly due to the low specificity or efficiency when they recognize and cleave DNA targets through protein-DNA interactions. In contrast, the recently developed CRISPR/Cas system is more convenient and efficient in mediating genome modification in different plants.

CRISPR/Cas genome editing system was adopted from bacteria and archaea’s adaptive immunity against viruses and plasmid (Wiedenheft et al. 2012). CRISPR/Cas9 system consists of a single endonuclease-Cas protein and a synthetic single-guide RNA (sgRNA) (Barrangou et al. 2007). The sgRNA contains a fusion of CRISPR RNA (crRNA) and *trans*-activating crRNA. It is divided into two classes based on the structure of the Cas protein complexes: Class 1 systems, including types I, III, and IV, and Class 2 systems, including types II, V, and VI. Class 1 uses multiprotein complexes to destroy foreign nucleic acids whereas Class 2 uses a single protein (Makarova et al. 2020). The most widely used Cas systems are based on Cas9 and Cas12a (Cpf1) nucleases, both of which contain a single protein effector and belong to Class 2 system (Ran et al. 2013; Makarova et al. 2020).

Beyond conventional DSB-mediated genome editing, recent developed gene editing systems such as base editor and prime editor could edit genomic DNA without DSBs. Base editor system, which includes cytosine base editors (CBEs) and adenine base editors (ABEs), contains a nicked Cas9 (nCas9) or catalytically inactivated Cas9 (dCas9), fused with single-stranded DNA (ssDNA) specific deaminase (Fig. 2D-E). These deaminases catalyze either C•G to T•A or A •T to G •C transitional changes based on the specific function of deaminases (Komor et al. 2016; Nishitani et al. 2016) (Fig. 2H-I). Prime editor contains two parts. One part is a fusion protein of Cas9 nickase (H840A) and reverse transcriptase, and the other part is prime editing guide RNA (pegRNA). The Cas9 nickase (H840A) recognize and break the non-target DNA strand. After reverse transcription with desired edit sequence on pegRNA as template, the edited DNA was synthesized (Fig. 2F). Prime editor has become an advanced system for precise base substitution, DNA deletion, or DNA insertion modifications (Fig. 2J) (Anzalone et al. 2019).

Among different genome editing technologies, CRISPR/Cas9 system, and it derived base editor and prime editor systems have become powerful tools for gene editing. Here, we mainly focus on the application of CIRPSR/Cas system as well as base editor in the field of economically important fruit crops (Table 1). We mainly describe the status of the application of these technologies in plant development and plant resistance. Subsequently, we highlight future perspective on applying genome editing for fruit crop improvement, in order to provide insights for future plant breeding in fruit crops.

**Table 1:** Recent advances of CRISPR/Cas genome editing in economically important fruit crops

Species	Promoter	Nuclease	Target Gene(s)	Target Trait(s)	Cas9 delivery method	Refence
*Fragaria vesca*	AtUBQ10	Cas9	*TAA1, ARF8*	Fruit development	Agrobacterium-mediated	(Zhou et al., 2018)
*Fragaria vesca*	35S	Cas9	*YUCCA10*	Fruit development	Agrobacterium-mediated	(Feng et al., 2019)
*Fragaria vesca*	35S	Cas9	*SEP3*	Flower and fruit development	Agrobacterium-mediated	(Pi et al., 2021)
*Fragaria vesca*	AtUBQ10	Cas9	*AGL62, ALG80*	Fruit development	Agrobacterium-mediated	(Guo et al., 2022)
*Fragaria vesca*	Ubi	nCas9 (D10A)	*bZIPs1.1*	Fruit sugar content	Agrobacterium-mediated	(Xing et al., 2020)
*Fragaria vesca*	Ubi	Cas9	*MYB10, CHS, PDS, UF3GT, F3H, LDOX*	Fruit coloration	Agrobacterium-mediated	(Xing et al., 2018)
*Fragaria vesca*	35S	Cas9	*LAM*	Plant architecture	Agrobacterium-mediated	(Feng et al., 2021a)
*Fragaria ananassa*	35S	Cas9	*TM6*	Flower and fruit development	Agrobacterium-mediated	(Martin-Pizarro et al., 2019)
*Fragaria ananassa*	35S	Cas9	*RAP*	Fruit coloration	Agrobacterium-mediated	(Gao et al., 2020)
*Cucumis sativus*	35S	Cas9	*WIP1*	Flower development	Agrobacterium-mediated	(Hu et al., 2017)
*Cucumis sativus*	35S	Cas9	*SPT,ALC*	Flower and fruit development	Agrobacterium-mediated	(Cheng et al., 2022)
*Cucumis sativus*	35S	Cas9	*HEC2*	Fruit development	Agrobacterium-mediated	(Wang et al., 2021e)
*Cucumis sativus*	35S	Cas9	*NS*	Fruit development	Agrobacterium-mediated	(Liu et al., 2022)
*Citrullus lanatus *	UBI	Cas9	*PSK1*	Resistance to *Fusarium oxysporum*	Agrobacterium-mediated	(Zhang et al., 2020b)
*Citrullus lanatus*	35S	Cas9	*WIP1*	Flower development	Agrobacterium-mediated	(Zhang et al., 2020a)
*Citrullus lanatus*	35S	Cas9	*PDS*	Albino phenotype	Agrobacterium-mediated	(Tian et al., 2017)
*Citrullus lanatus*	35S	Cas9	*COMT1*	Fruit quality, abiotic stress	Agrobacterium-mediated	(Chang et al., 2021)
*Citrullus lanatus*	35S	Cas9	*NAC68*	Fruit sugar content	Agrobacterium-mediated	(Wang et al., 2021b)
*Citrullus lanatus*	35S	Cas9	*BG1*	Seed development	Agrobacterium-mediated	(Wang et al., 2021c)
*Cucumis melo*	PcUbi4-2	Cas9	*ROS1, CTR1-like*	Fruit ripening	Agrobacterium-mediated	(Giordano et al., 2022)
*Vitis vinifera*	35S	Cas9	*IdnDH*	Fruit quality	Agrobacterium-mediated	(Ren et al., 2016)
*Vitis vinifera*	PcUbi4-2	Cas9	*PDS*	Albino phenotype	Agrobacterium-mediated	(Nakajima et al., 2017)
*Vitis vinifera*	VvUBQ2	Cas9	*TMT1, TMT2, PDS*	Fruit sugar accumulation; Albino phenotype	Agrobacterium-mediated	(Ren et al., 2021)
*Vitis amurensis*	35S	Cas9	*PAT1*	Cold response	Agrobacterium-mediated	(Wang et al., 2021d)
*Vitis vinifera*	35S	Cas9	*CCD8*	Shoot branching	Agrobacterium-mediated	(Ren et al., 2020)
*Vitis vinifera*	35S	Cas9	*PR4b*	Defense against the downy mildew	Agrobacterium-mediated	(Li et al., 2020a)
*Vitis vinifera*	35S	Cas9	*MLO3, MLO4*	Defense against the powdery mildew	Agrobacterium-mediated	(Wan et al., 2020)
*Vitis vinifera*	35S	Cas9	*AGL104*	Flower, fruit and seed development	Agrobacterium-mediated	(Sun et al., 2020b)
*Citrus sinensis*	35S	Cas9	*PDS*	Albino phenotype	Agrobacterium-mediated	(Jia and Wang, 2014)
*Citrus sinensis*	35S	Cas9	*PDS*	Albino phenotype	Agrobacterium-mediated	(Dutt et al., 2020)
*Citrus sinensis*	35S, Yao	LbCas12a	*PDS, L0B1*	Resistant to citrus canker	Agrobacterium-mediated	(Jia et al., 2019)
*Citrus sinensis*	35S	Cas9	*NPR3*	Systemic acquired resistance (SAR)	Cationic lipid transfection with or without PEG	(Mahmoud et al., 2022)
*Citrus sinensis*	CmYLCV	PC-ABE8e	*LOB1*	Resistant to canker	Agrobacterium-mediated	(Huang et al., 2022)
*Citrus paradise*	CmYLCV	nCas9	*ALS*	resistant to the herbicide	Agrobacterium-mediated	(Huang et al., 2022)
*Malus prunifolia*	35S	Cas9	*PDS*	Albino phenotype	Agrobacterium-mediated	(Nishitani et al., 2016)
*Malus domestica *	PcUbi4-2	nCas9	*ALS, PDS*	Resistance to chlorsulfuron and albino	Agrobacterium-mediated	(Malabarba et al., 2020)
*Malus domestica*	AtUBQ10	Cas9	*DIPM4*	Resistance to fire blight disease	Agrobacterium-mediated	(Pompili et al., 2020)
*Malus domestica*	AtUBQ10	LbCas12a	*PDS*	Albino phenotype	Agrobacterium-mediated	(Schropfer and Flachowsky, 2021)
*Malus domestica*	PcUbi4-2	Cas9	*PDS, TFL1*	Albino phenotype; early flowering	Agrobacterium-mediated	(Charrier et al., 2019)
*Malus sieverii*	ZmUbi, 35S	Cas9	*PDS*	Albino phenotype	Agrobacterium-mediated	(Zhang et al., 2021)
*Malus domestica*	35S	Cas9	*CNGC2*	Resistance to *B. dothidea*	Agrobacterium-mediated	(Zhou et al., 2020a)
*Malus domestica*	35S	Cas9	*MKK9*	Fruit color	Agrobacterium-mediated	(Sun et al., 2022)
*Solanum lycopersicum*	35S	Cas9	*SHR*	Root development	Agrobacterium-mediated	(Ron et al., 2014)
*Solanum lycopersicum*	35S	Cas9	*AGO7*	Leaf morphology	Agrobacterium-mediated	(Brooks et al., 2014)
*Solanum lycopersicum*	35S, AtUBQ	Cas9	*PDS, PIF4*	Albino phenotype, light signal transduction	Agrobacterium-mediated	(Pan et al., 2016)
*Solanum lycopersicum*	35S	Cas9	*ARF4*	Plant growth, resistance to abiotic stress	Agrobacterium-mediated	(Bouzroud et al., 2020)
*Solanum lycopersicum*	35S	Cas9	*SRM1-like*	Leaf development	Agrobacterium-mediated	(Tang et al., 2022)
*Solanum lycopersicum*	35S	nCas9 (D10A)	*DELLA, ETR1*	Hormone signaling	Agrobacterium-mediated	(Shimatani et al., 2017)
*Solanum lycopersicum*	35S	Cas9	*SP,* *SP5G, ER*	Plant architecture	Agrobacterium-mediated	(Kwon et al., 2020)
*Solanum lycopersicum*	35S	Cas9	*BOP*	Inflorescence development	Agrobacterium-mediated	(Xu et al., 2016)
*Solanum lycopersicum*	35S	Cas9	*DOF9*	Inflorescence and flower development	Agrobacterium-mediated	(Hu et al., 2022)
*Solanum lycopersicum*	Ubi	Cas9	*ORRM4*	Fruit ripening	Agrobacterium-mediated	(Yang et al., 2017)
*Solanum lycopersicum*	Ubi	Cas9	*LncRNA1459*	Fruit ripening	Agrobacterium-mediated	(Li et al., 2018a)
*Solanum lycopersicum*	35S, PcUbi4-2	Cas9	*IAA9*	Fruit development	Agrobacterium-mediated	(Ueta et al., 2017)
*Solanum lycopersicum*	PcUbi4-2	Cas9	*GAD2, GAD3*	Fruit quality	Agrobacterium-mediated	(Nonaka et al., 2017)
*Solanum lycopersicum*	35S	Cas9	*PSY1*	Fruit color	Agrobacterium-mediated	(Filler Hayut et al., 2017)
*Solanum lycopersicum*	Ubi	Cas9	*MIR164A*	Fruit ripening and chloroplast development	Agrobacterium-mediated	(Lin et al., 2022b)
*Solanum lycopersicum*	PcUbi4-2	Cas9	*KIX9; SlKIX8*	Plant organ size	Agrobacterium-mediated	(Swinnen et al., 2022)
*Solanum lycopersicum*	35S	Cas9	*CRCa*	Floral meristem determinacy	Agrobacterium-mediated	(Castaneda et al., 2022)
*Solanum lycopersicum*	35S	Cas9	*CLV3, WOX9, TFL1,*	Floral organ number, fruit size	Agrobacterium-mediated	(Rodriguez-Leal et al., 2017)
*Solanum lycopersicum*	35S	Cas9	*ENO*	Fruit size	Agrobacterium-mediated	(Yuste-Lisbona et al., 2020)
*Solanum lycopersicum*	Ubi	Cas9	*MYC2*	Plant development, disease resistance	Agrobacterium-mediated	(Shu et al., 2020)
*Solanum lycopersicum*	35S	Cas9	*AGL6*	Fruit development	Agrobacterium-mediated	(Klap et al., 2017)
*Solanum lycopersicum*	35S	Cas9	GGP1	Fruit quality	Agrobacterium-mediated	(Deslous et al., 2021)
*Solanum lycopersicum*	35S	Cas9	*INVINH1,VPE5*	Fruit sugar content	Agrobacterium-mediated	(Wang et al., 2021a)
*Musa acuminata*	35S	Cas9	*RAS-PDS*	Albino phenotype	Agrobacterium-mediated	(Kaur et al., 2018)
*Musa acuminata*	ZmUbi1, 35S	Cas9	*PDS*	Albino phenotype	Agrobacterium-mediated	(Naim et al., 2018)
*Musa acuminata*	Ubi	Cas9, LbCas12a	*PDS*	Albino phenotype	PEG mediated RNP	(Wu et al., 2020)
*Musa acuminata*	35S	Cas9	*PDS*	Albino phenotype	Agrobacterium-mediated	(Ntui et al., 2020)
*Musa acuminata*	35S	Cas9	*LCYε*	Fruit quality	Agrobacterium-mediated	(Kaur et al., 2020)
*Musa acuminata*	Ubi	Cas9	*GA20OX2*	Plant height	Agrobacterium-mediated	(Shao et al., 2020)
*Musa acuminata*	Ubi	Cas9	*ACO1*	Ethylene production; Fruit shelf	Agrobacterium-mediated	(Hu et al., 2021)
*Musa acuminata*	Ubi	Cas9	*PDS*	Albino phenotype	Protoplast transformation	(Zhang et al., 2022)
*Musa acuminata*	35S	Cas9	*CCD4*	Carotenoids metabolism	Particle bombardment	(Awasthi et al., 2022)
*Actinidia chinensis*	35S	Cas9	*PDS*	Albino phenotype	Agrobacterium method	(Wang et al., 2018b)
*Actinidia chinensis*	35S	Cas9	*FLCL*	Flower development	Agrobacterium method	(Voogd et al., 2022)
*Actinidia chinensis*	35S	Cas9	*SyGl, CEN4*	Flower development	Agrobacterium method	(Varkonyi-Gasic et al., 2021)
*Actinidia chinensis*	Ubi, 35S	Cas9	*CEN, CEN4*	Plant stature, early flowering	Agrobacterium method	(Varkonyi-Gasic, et al., 2019)

## Application of genome editing in fruit crops

### Plant development

Fruit crops are major economic crops in many countries or regions in the world and are of great importance to the global economy and human nutrients. Fleshy fruits are divided into climacteric and non-climacteric types based on different respiratory features and ethylene biosynthesis rates during fruit ripening (Giovannoni 2001). In climacteric fruits such as apple, banana, tomato, kiwifruit and peach, the gaseous hormone ethylene is required for fruit ripening (Brumos 2021). Whereas in non-climacteric fruits such as strawberry, grape, watermelon, cucumber and citrus, fruit ripening is mainly regulated by abscisic acid (ABA), and ethylene is dispensable for the ripening process (Giovannoni 2001; Cherian et al. 2014). Many efforts have been made to optimize plant genome editing systems to improve fruit quality in climacteric fruits and non-climacteric fruits.

#### Non-climacteric fruits (strawberry, grape, watermelon, cucumber, citrus)

##### Strawberry

As a versatile experimental plant system, wild diploid strawberry *Fragaria vesca* has been developed as a model for non-climacteric plant study due to its short life-cycle, low genome complexity (2n = 14), and feasibility of transformation (Shulaev et al. 2011). The CRISPR/Cas9 system has been applied in modifying plant architecture in strawberry. Axillary bud development is a major factor that impacts strawberry growth and production. A GRAS transcription factor *LOSS OF AXILLARY MERISTEMS* (*LAM*) has been identified to regulate runner formation in a way sequentially with Gibberellic acid (GA). CRISPR/Cas9 mediated *lam* knock-out mutants exhibited fewer runners than wild types (Feng et al. 2021a).

**Fig. 3:**
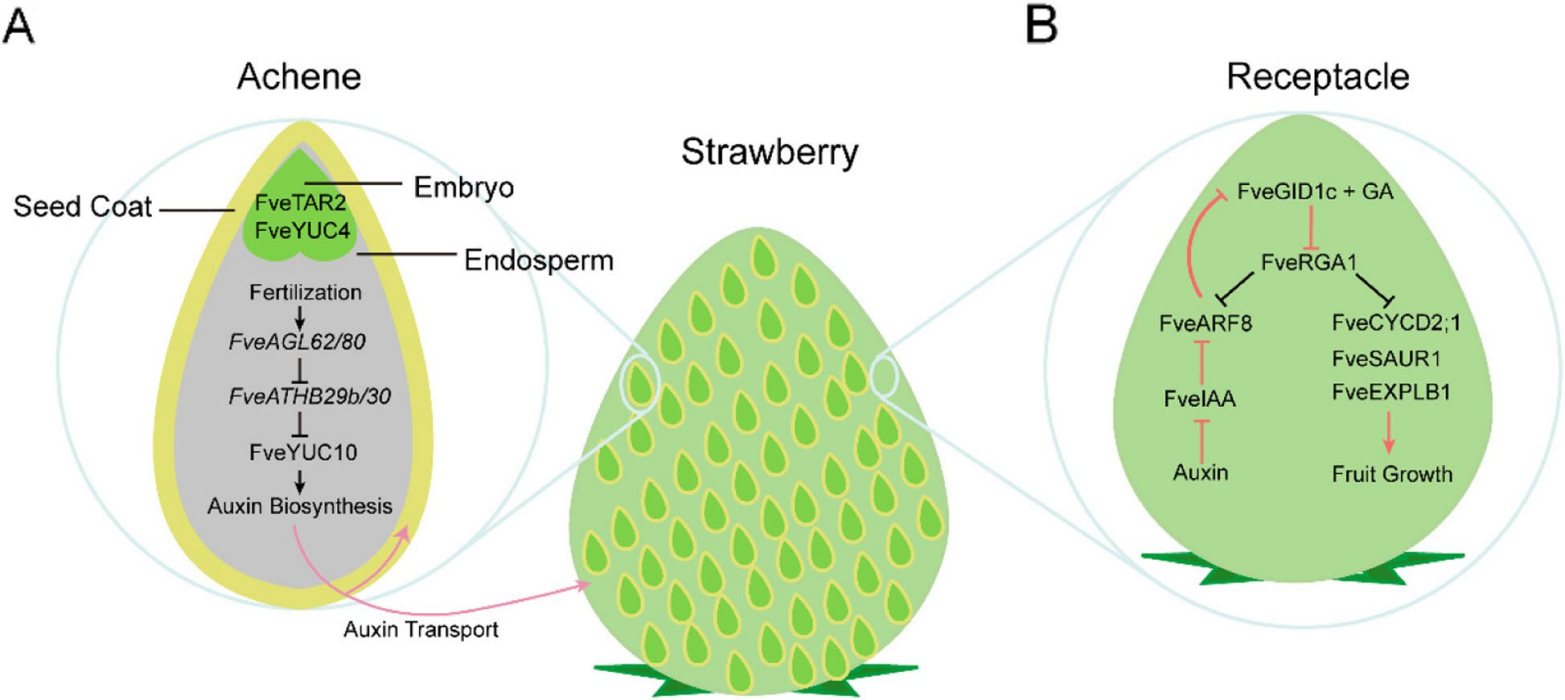
Strawberry fruit structure and a model illustrating the regulatory pathway during fruit initiation and growth. **A** Double fertilization promotes biosynthesis of auxin and GA in the seed. Auxin and GA can stimulate receptacle development after being transported to the receptacle. **B** A diagram of receptacle illustrating the regulatory mechanism of fruit set. *FveRGA1* is shown as a central player. The red lines indicate regulatory actions post-fertilization. Positive (arrows) or negative (bar) regulations are indicated. Adapted from (Feng et al. 2019; Zhou et al. 2021; Guo et al. 2022)

CRISPR/Cas9 system has been widely employed in investigating the mechanism of fruit development. The strawberry fruit is derived from the receptacle, and it offers a great system to investigate signaling communication between seeds and fruits during fruit set (Hollender et al. 2012; Kang et al. 2013a; Cappelletti et al. 2015; Härtl et al. 2017). The strawberry fruit set is initiated by auxin and GA which were produced in the achene (Nitsch 1950; Kang et al. 2013a). Transcriptome analyses revealed that both auxin and GA biosynthesis genes are highly expressed in the seeds, such as *YUCCA* (*YUC*), *GA 20-oxidases (GA20ox)*, and *GA3-oxidases* (*GA3ox*), while the genes required for perception (*TRANSPORT INHIBITOR RESPONSE 1* and GIBBERELLIN INSENSITIVE DWARF1) and signaling (*AUXIN RESPONSE FACTORS* and *REPRESSOR OF GA1*) of both hormones are more highly expressed in the receptacles (Kang et al. 2013a) (Fig. 3). However, the molecular basis for phytohormone-mediated fruit initiation was not fully elucidated until the stable gene-edited strawberry plants were generated by the first reported CRISPR/Cas9 system (JH4-JH19) developed in strawberries (Zhou et al. 2018). In this system, the *Arabidopsis U6* promoter (*AtU6–26*) and *Fragaria vesca FveU6* promoter were tested for editing efficiency, with *AtU6–26* showing slightly higher editing efficiency in Arabidopsis protoplasts. However, both *AtU6–26* and *FveU6–2* promoters exhibited a similar mutation efficiency (T0: 49–75%) in stable transgenic *F. vesca* lines, suggesting that the *U6* promoters from different dicots may share conversed functions. The resulting *arf8* homozygous mutants showed faster growth of seedling and increased number of petals in the single flower, as well as increased width and height of fruit compared to the wild type (Fig. 3) (Zhou et al. 2018; Zhou et al. 2021). By using CRISPR/Cas9, a knock-out mutation at the *FveYUC10* locus was generated. The knockout of *FveYUC10* reduced free auxin content, but there no phenotype changes were observed, probably due to the functional redundancy of *YUC* genes (Feng et al. 2019). The auxin-dependent fruit ripening mechanism has been further illustrated by using the strawberry-specific CRISPR/Cas9 system (JH4-JH19) targeting the *AGAMOUS-LIKE62* (*FveAGL62*) gene (Guo et al. 2022). The *agl62* mutants showed reduced auxin biosynthesis and failed to initiate fruit (Fig. 3). In addition to auxin, certain A, B, C, and E-type MADS-box genes regulate the initiation and development of fleshy fruits. Recently, it has been speculated that certain MADS-box genes of the B and E class could determine fleshy fruit identity or development (Liu et al. 2020b). A CRISPR/Cas9 tool was used to knock out a B class MADS-box gene *TOMATO MADS BOX GENE6* (*TM6)* in cultivated strawberry *Fragaria ananassa*, which led toabnormal pollen and aborted fruits (Martin-Pizarro et al. 2019). This was the first report of successfully apply the CRISPR/Cas9 system in cultivated octoploid strawberry. Another study revealed the role of the E-Class MADS-box gene, *SEPALLATA3* (*FveSEP3)*, in strawberry fruit development through CRISPR/Cas9-mediated genome editing. The CRISPR/Cas9-edited *fvesep3* mutant strawberry produced parthenocarpic fruits, which is a preferred trait in strawberry fruit breeding (Liu et al. 2020b; Pi et al. 2021). Applying the CRISPR/Cas9 system to illustrate molecular mechanisms of fruit development has provided candidate gene resources for molecular breeding in strawberries and other non-climacteric fruit crops.

CRISPR/Cas9 and other genome editing tools were also applied to strawberries for fruit quality improvement. Strawberry has been a pioneer fruit crop in implementing the CRISPR/Cas9-derived base editor system to fine-tune its sugar content. Using a highly efficient plant CBE base editor system (A3A-PBE) with the APOBEC3A deaminase, the upstream open reading frame (uORF) of transcription factor gene *FvebZIPs1.1* was targeted. Sixty-six T0 transgenic plants were obtained, and 60 out of 66 were homozygous and biallelic, accounting for 90.9% of the genetic changes observed. The gene-edited strawberries showed higher sugar content than controls, and more importantly the edited plants were transgene-free (Xing et al. 2020). This is the first report to apply the base editor system to target the uORF region of target genes in strawberries, offering a powerful tool to achieve gene activation without introducing transgenic elements, hence, this method endows a bright future in quantitative trait improvement and germplasm innovation in strawberry and other fruit crops. Fruit color is determined by anthocyanin content. Previous work demonstrated that *R2R3 MYB TRANSCRIPTION FACTOR 10* (*FveMYB10*) is a key transcription factor for strawberry fruit color formation which functions by regulating the anthocyanin biosynthesis gene *CHALCONE SYNTHASE* (*FveCHS*) (Medina-Puche et al. 2014). With a high-efficient CRISPR/Cas9 system, *FveMYB10* and *FveCHS* were targeted via agroinfiltration-mediated transient transformation in both octoploid and diploid strawberry fruits, leading to a partially delayed anthocyanin accumulation (Xing et al. 2018). Recently, it was found that *FveMYB10* and *FveCHS1* were phosphorylated and transcriptionally repressed by *MITOGEN-ACTIVATED PROTEIN KINASE3* (*FveMAPK3*) under cold conditions. In this study, the conventional CRISPR/Cas9 constructs were used to generate *FveMAPK3* mutants. However, only two heterozygous mutants were identified among 58 transgenic diploid strawberry lines, and unexpected changes in ploidy (tetraploid) were identified. It has offered insights into the molecular basis underlying low temperature-mediated fruit coloring delay and suggested a novel strategy to generate cold-tolerance strawberry germplasm (Mao et al. 2022). The CRISPR/Cas9 system was also applied in cultivated strawberries to generate desired white fruit phenotype. For example, the anthocyanins transport gene *REDUCED ANTHOCYANINS IN PETIOLES (RAP)* was targeted in octoploid strawberry ‘NingYu’. Through CRISPR/Cas9 system, six copies of the anthocyanins transport gene *REDUCED ANTHOCYANINS IN PETIOLES (RAP)* were simultaneously knocked out in the cultivated octoploid strawberries, in which it is hard to edit all homologous alleles. The edited *rap* mutants developed white fruits instead of red fruits in the cultivated strawberry. This work offered a promising candidate gene for fruit-color breeding in cultivated strawberries (Gao et al. 2020).

##### Cucumber

Cucumber (*Cucumis sativus* L*.*), consumed as both vegetable and fruit, is cultivated in about 2.3 million hectare area across 140 countries in the world (Liu et al. 2022). Cucumber fruit production is closely correlated with flowers, which contain unisexual male or female flowers. Gynoecious cucumber flower has been a preferred trait for breeding, in this way the hand emasculation could be avoided in gynoecious lines (Hu et al. 2017). A C2H2 zinc-finger transcription factor *WIP1* has been reported to regulate gynoecism in *Cucumis melo* (Martin et al. 2009). The CRISPR/Cas9 system has been applied in cucumber to generate genome-engineered gynoecious flowers by targeting *CmWIP1* genes. In this system, endogenous *CsU6–1* promoter was applied to drive sgRNA expression. The mutation rate on the *CsWIP1* locus reached 64.3%, and homozygous transgene-free *cswip1* mutants bore seven times more female flowers than the wild type (Hu et al. 2017). The CRISPR/Cas9 system has also been used to target a basic Helix-Loop-Helix (bHLH) gene, *Cucumis sativus SPATULA* (*CsSPT)*, which was found to act redundantly with *ALCATRAZ* (*CsALC*) to confer female sterility (Cheng et al. 2022). These reports provide valuable targets for molecular breeding of cucumber varieties with desired traits.

CRISPR/Cas9-mediated gene-edited fruits with desirable quality traits are in great demand. Cucumber has a special warty trait, consisting of spines and tubercules that greatly affect fruit appearance and market value. *CsHEC2*, a basic Helix-Loop-Helix (bHLH) gene regulating wart formation, was edited with CRISPR/Cas9. Two homozygous T2 *cshec* mutant lines exhibited reduced wart density (Wang et al. 2021e). A more detailed mechanism of wart development was investigated by editing genes such as *Csa2G264590* (Liu et al. 2022). CRISPR/Cas9-mediated *Csa2G264590* mutation led to an increase in spine number over ten times that of the wild type (Liu et al. 2022). These works demonstrated that the CRISPR/Cas9 system has advanced improvements in cucumber fruit quality and is of great value for cucumber breeding.

##### Watermelon

Watermelon (*Citrullus lanatus* (Thunb*.*) Matsum. & Nakai) is the second species in the Cucurbitaceae family with successful genome editing (Zhou et al. 2020b), and researchers have mainly focused on editing genes involved in watermelon flower sex determination. Genome editing of a C2H2 zinc finger transcription factor *ClWIP1* led to the formation of gynoecious watermelon, with female and hermaphroditic flowers observed in homozygous T1 mutants (Zhang et al. 2020a).

The CRISPR/Cas9 system has also been applied for watermelon fruit quality improvement. Sugar content is mediated by NAC (NAM, ATAF1/2, and CUC2) transcription factors. CRISPR/Cas-mediated genome editing of the *ClNAC68* resulted in decreased contents of fructose, glucose, and sucrose accumulation (Wang et al. 2021b). Moreover, Melatonin (*N*-acetyl-5-methoxytryptamine) is identified as a key bioactive molecule involved in many processes in plants and animals (Zhang et al. 2015b). Caffeic acid O-methyltransferase gene *ClCOMT1* plays a key role in melatonin biosynthesis (Kang et al. 2013b). CRISPR/Cas9 mediated *clcomt1* mutation led to decreased melatonin contents in watermelon calli (Chang et al. 2021). In addition, the CRISPR/Cas system has been used to target the ABA hydrolyzation β-glucosidase (*BG*) gene (Wang et al. 2021c) to generate transgene-free *bg1* mutants with reduced seed size and weight.

##### Grape

Grape (*Vitis vinifera* L.), a member of the Vitaceae family, is one of the most economically important berry fruit and is the major source of wine production worldwide (Gupta et al. 2020). CRISPR/Cas9 was applied in grapes to improve plant morphology and architecture several years ago. CRISPR/Cas9 was adopted to edit the *PHYTOENE DESATURASE (VvPDS*) gene in grapes by using an *Arabidopsis AtU6* promoter to drive the sgRNA-*VvPDS* expression cassette. A Cas9 expression cassette and the synthetic sgRNA were transformed into grape embryonic calli, leading to albino leaves in the regenerated plants (Nakajima et al. 2017). Subsequently, CRISPR/Cas9 was applied to target the strigolactones biosynthesis gene *CAROTENOID CLEAVAGE DIOXYGENASE 8* (*VvCCD8)*. In this study, the mutation efficiency reached to 66.7%. The acquired *vvccd8* mutant lines exhibited increased shoot branches (Ren et al. 2020). This effort highlights essential candidate genes for grape architecture improvement using CRISPR/Cas9 tools.

In addition, CRISPR/Cas9 has been applied to grapes to study fruit development and quality. Grapes accumulate L-tartaric acid, which is a plant-derived metabolite and its oxidation results in vitamin C catabolism (DeBolt et al. 2006). CRISPR/Cas9 was first applied to grapes to target the L-idonate dehydrogenase gene (*IdnDH,* LOC100232980), a tartaric acid biosynthesiss gene. In this study, the sgRNA expression cassette and Cas9 protein were driven by the *Arabidopsis* U6 promoter, *AtU6*, and 35S promoter, respectively. Three of the six *idndh* transgenic lines were obtained, showing decreased tartaric acid content compared to controls (Ren et al. 2016). This study presents a new strategy to improve fruit quality by editing the toolkit genes in grapes and other perennial fruit plants.

##### Citrus

Citrus is one of the most widely grown fruit crops in the world, the global production of citrus has increased from 42.1 million tons in 1971 to 161 million tons in 2021. Citrus breeding is challenging due to its polyembryonic seeds, pollen incompatibility, and recalcitrant to agroinfiltration (Talon and Gmitter Jr. 2008; Zhu et al. 2020). In 2014, a *Xanthomonas citri* subsp. *citri* (*Xcc*)-facilitated agroinfiltration method was developed in Valencia sweet orange and the *CsPDS* gene was successfully knocked out. This transiently expressed CRISPR/Cas9 system resulted in mutation rates ranging from 3.2 to 3.9% and highlighted its potential to perform functional study and germplasm innovation in these long-lifespan shrub fruit crops (Jia and Wang 2014). Recently, a more efficient genome editing system, namely tRNA-mediated or Csy4-mediated multiplex genome editing using citrus *U6* promoter, was shown to promote the mutation rate up to 44.4% (Huang et al. 2020). The same group further improved the CRISPR/Cas9 system using different strategies, including adopting *Cestrum yellow leaf curling virus* (*CmYLCV*) and using *Citrus sinensis* ubiquitin (*CsUBI*) promoter to drive Cas9 expression together with an optimized culture temperature (Huang et al. 2021). These novel genome editing systems present new opportunities to perform functional studies in citrus.

#### Climacteric fruits (apple, tomato, banana, kiwifruit)

##### Apple

Apple is a major fruit consumed worldwide with a high value of nutrients and antioxidants. Efficient genome editing in apple was first reported in rootstock ‘JM2’, which demonstrated the successful application of CRISPR/Cas9 in apple by targeting the *PDS* gene (Nishitani et al. 2016). In a recent study, the efficiency of CRISPR/Cas9-mediated genome editing was compared between two systems; one consisted of Cas9 driven by the 35S promoter and sgRNA driven *by* the *AtU6–26* promoter, while the other consisted of Cas9 driven by the maize ubiquitin promoter and sgRNA driven by the *AtU3d* promoter. Both systems work, but the first system was proven more efficient in achieving consistency in genome editing (Zhang et al. 2021). To increase the efficiency of the CRISPR/Cas9 system, the promoter driving the sgRNA cassette was further optimized by changing to *MdU3* and *MdU6* promoters from ‘Gala’. The optimized system led to a mutation efficiency of 84% for *MdPDS* and 90% for *TERMINAL FLOWER 1* (*MdTFL1)* in apples (Charrier et al. 2019). In addition, the *MdU6* promoter-driving system was used for targeting *MITOGEN ACTIVATED PROTEIN KINASE KINASE 9* (*MdMKK9*) and yielded two different mutations at the target site among four positive calli lines, leading to a reduced anthocyanin content compared to the wild type (Sun et al. 2022). Another genome editing system composed of pHDE-35S-Cas9-mCherry-UBQ cassette and sgRNA driven by *MdU6* promoter also achieved gene mutation in all ten sequenced kanamycin-resistant calli (Zhou et al. 2020a).

##### Tomato

Tomato (*Solanum lycopersicum* L.) was regarded as a model climacteric fruit for investigating fruit development and ripening mechanisms (Zhou et al. 2020b). CRISPR/Cas9 was first applied in tomato to target the *SHORT ROOT* (*SlSHR)* gene using the transient hairy root transformation method (Ron et al. 2014). The first CRISPR/Cas9-induced stable transgenic tomato mutation lines were produced by targeting tomato *ARGONAUTE7* (*SlAGO7*)*,* with a mutagenesis efficiency as high as 48% (Brooks et al. 2014). Transgenic *slago7* mutants showed altered radialized leaflets, similar to the previously reported *slago7* alleles (Yifhar et al. 2012). After that, CRISPR/Cas9 has been successfully used to change plant architecture and leaf morphology. Many genes involved in plant development have been edited using the CRISPR/Cas9 or CBE systems in tomatoes, such as *SlPDS* (Pan et al. 2016), DELLA-encoding *PROCERA* gene (Tomlinson et al. 2019), *SALT-RELATED MYB1*-like (*SlSRM1-like*) gene (Tang et al. 2022), and *AUXIN RESPONSE FACTOR 4* (*SlARF4*) (Bouzroud et al. 2020). For example, the CBE base editor system has been applied to target the phytohormone signaling genes, *DELLA* or *ETHYLENE RESPONSE1* (*ETR1*), in the tomato cultivar ‘Micro-Tom’. By using this system, heritable DNA substitutions and transgene-free plants were generated (Shimatani et al. 2017). Later, a novel DELLA dwarfing tomato allele was generated using a CRISPR/Cas9 construct targeting the conserved DELLA motif (Tomlinson et al. 2019).

CRISPR/Cas9 could be applied to generate a series of desired quantitative traits for plant architecture improvement. The promoter of the homeobox gene, *COMPOUND INFLORESCENCE* (homolog of *Arabidopsis WUSCHEL-RELATED HOMEOBOX 9, WOX9*), was targeted by CRISPR/Cas9. In total, 326 sensitized F1 plants were obtained, and 28% of them exhibited different branching phenotypes (Rodriguez-Leal et al. 2017). Similarly, the promoter of *SELF PRUNING* (*SP*), homolog of *Arabidopsis TERMINAL FLOWER 1* (*TFL1*), was edited to generate a series of different plant architectures (Rodriguez-Leal et al. 2017). Another example is the flowering repressor gene *SP* and *ERECTA* (*ER*). CRISPR/Cas9-induced *slsp/slsp5g/sler* multiplexed mutations enhanced plant compactness and increased yield (Kwon et al. 2020). These genes may serve as good candidates for genome editing-mediated tomato fruit breeding in the future.

In addition, the CRISPR/Cas9 system has been applied to regulate flowering in tomatoes. It is known that tomato multiflowered inflorescences are regulated by the transcriptional complex consisting of TERMINATING FLOWER (TMF) and BLADE-ON-PETIOLE (BOP). A CRISPR/Cas9-mediated knockout of *SlBOP* led to the transition of sympodial growth from inflorescences to single flowers (Xu et al. 2016). In contrast, CRABS CLAW (CRC) paralogues positively regulate floral meristem (FM) determinacy and ensure the proper formation of flowers and fruits. The CRISPR/Cas9 mediated *SlCRCa* mutants formed indeterminate FM (Castaneda et al. 2022). Flower development is negatively regulated by the C2H2-type zinc-finger-like transcription factor *DNA-BINDING WITH ONE ZINC FINGER 9* (*DOF9*). *Sldof9* knockout mutants produced more flowers per inflorescence and generated more occasional flower organ alterations (Hu et al. 2022). These results indicate a complex regulation mechanism in FM determinacy.

**Fig. 4:**
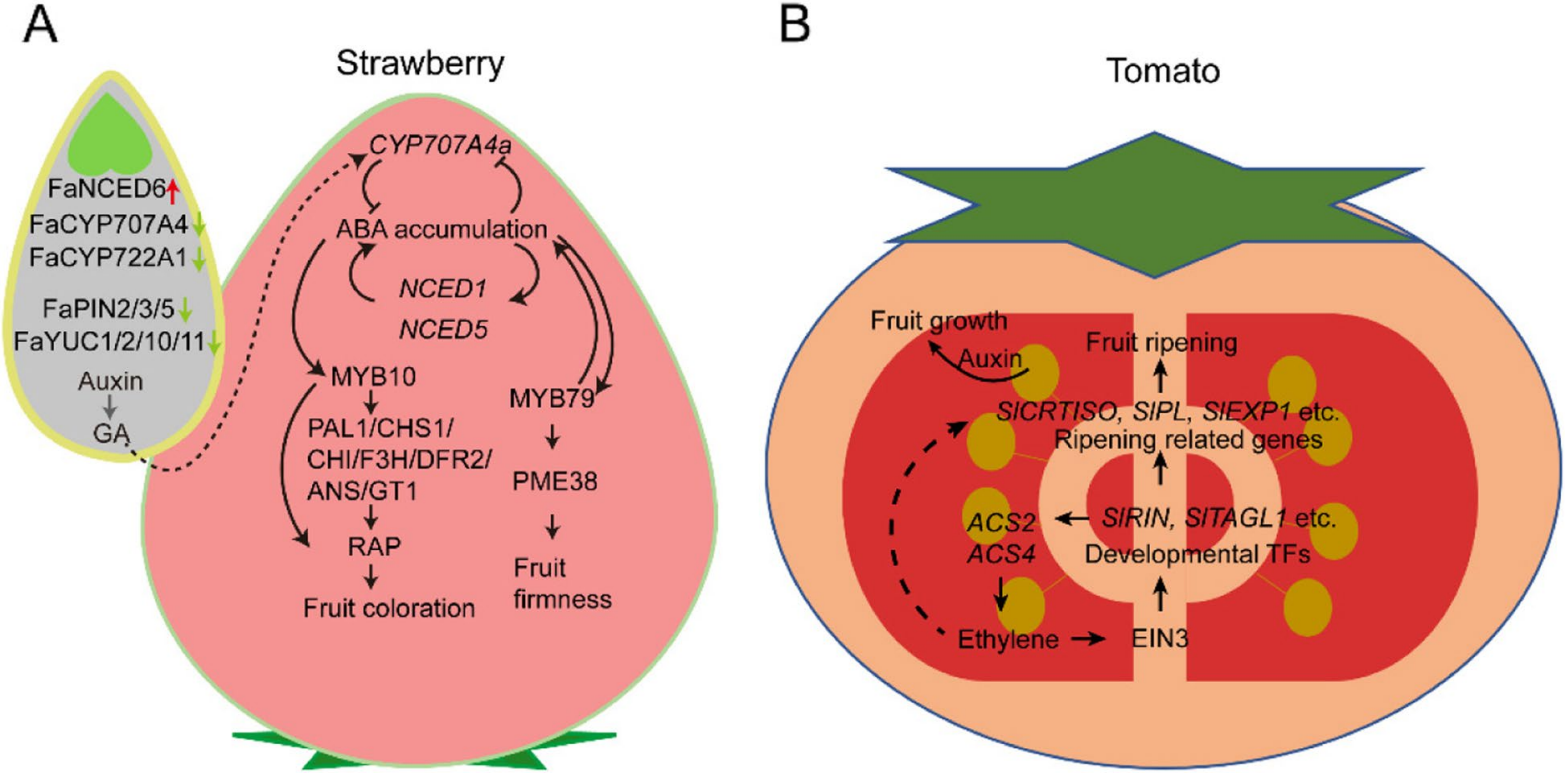
The interaction among ABA, auxin, and GA in regulating strawberry and tomato fruit development. **A** A diagram of strawberry fruit illustrating the regulatory mechanism during fruit ripening. In the achene, the expression of ABA biosynthesis gene (*FaNCED6*) increases, and the expression of ABA metabolism gene (*FaCYP707A4*, *FaCYP722A)* decreases, which resulted in a high ABA level. On the other hand, the expression of *FaPIN2/3/5* and *FaYUC1/2/10/11* decreases, which resulted in a reduced auxin and GA level. The feedforward loop of ABA level was activated, and the ripening related transcription factors (*MYB79*, *MYB10*) were activated to ensure fruit firmness and anthocyanin biosynthesis. As fruit ripens, JA level accumulates to ensure flavor formation. **B** In tomato, seeds are the site of auxin production. Auxin was also transported to the surrounding tissues to stimulate fruit growth. However, during fruit ripening, *SlEIN3* and *SlTAGL1,* activate ethylene production and form a positive feedback circuit. SlEIN3 and SlTAGL1 form a complex and promote fruit ripening by regulating transcription factors *SlCRTISO*, *SlPL*, *SlEXP1*, *SlCEL2* etc. On the other hand, the expression of *SlCRTISO*, *SlPL*, *SlEXP1* and *SlCEL2* is also related to ethylene production. However, whether there is a direct regulation is not clear. Adapted from (Kang et al. 2013a; Liao et al. 2018; Cao et al. 2020; Chen et al. 2020; Li et al. 2022)

Furthermore, CRISPR/Cas genome editing has been employed to study the molecular basis of fruit initiation. Fruit size enlargements and an increase in fruit production could be achieved by editing the negative or positive regulators of fruit development. For example, *YABBY2b* is involved in tomato fruit locule number formation (Dai et al. 2007; Cong et al. 2008), and *yabby2b* knockout mutants created by CRISPR/Cas9 exhibited reduced plant height, flower size, and fruit size (Sun et al. 2020a). Similarly, CRISPR/Cas9-mediated *EXCESSIVE NUMBER OF FLORAL ORGANS* (*ENO*) edited mutants produced larger multilocular fruits and more branched inflorescences (Yuste-Lisbona et al. 2020). Complex formed by PEAPOD (PPD) and KINASE-INDUCIBLE DOMAIN INTERACTING (KIX) could regulate plant organ size and shape. *kix8* and *kix9* mutants generated by CRISPR/Cas9 produced enlarged fruits and enlarged dome-shaped leaves (Swinnen et al. 2022). The CRISPR/Cas9 system has been employed to generate parthenocarpic tomato fruits, which is a desired agronomic trait since in this way fruit initiation is independent of fertilization and is less affected by environmental factors (Molesini et al. 2020). For instance, a CRISPR/Cas9 system-mediated *AGAMOUS LIKE 6* (*SlAGL6*) mutant produces facultative parthenocarpic tomato fruits and also promotes fruit production under heat-stress (Klap et al. 2017). A CRISPR/Cas9-induced somatic mutation of *AUXIN-INDUCED 9* (*SlIAA9*) caused significant changes in leaf shapes and seedless fruits (Ueta et al. 2017). These results offer numerous genomic resources for fruit crop breeding through a genome editing system (Fig. 4).

CRISPR/Cas9 has been widely applied to investigate tomato fruit ripening, which is regulated by transcriptional factors, such as *NO APICAL MERISTEM 1* (*SlNAM1*), *RIPENING INHIBITOR (RIN)*, *NON-RIPENING* (*SlNOR*), and *FRUITFULL* (*SlFUL1*). For a long time, these transcription factors have been acknowledged as master regulators of the ripening process (Vrebalov et al. 2002; Kitagawa et al. 2005; Klee and Giovannoni 2011). Their function and signaling pathway during fruit ripening were further investigated on gene-knockout mutants generated by CRISPR/Cas9 (Ito et al. 2015; Ito et al. 2017). For instance, *RIN* knock-out mutant plants only displayed delayed-ripening phenotypes, and *RIN*, *NOR*, and *COLORLESS NON-RIPENING* (*CNR*) are partially redundant in regulating tomato fruit ripening (Ito et al. 2017; Gao et al. 2019; Wang et al. 2019c; Ito et al. 2020; Wang et al. 2020). Small RNAs can also regulate tomato fruit ripening (Seymour et al. 2008). It has been demonstrated that transcriptional factors *SlNAM2* and *SlNAM3* were targeted for mutation by pYLCRISPR/Cas9Pubi-H system, resulting in the *slmir164a* edited mutants with earlier ripening phenotype (Lin et al. 2022b). Besides, the tomato fruit ripening process could be delayed by targeting RNA editing factors or lncRNAs. For example, the tomato RNA editing factor (*SlORRM4*) was targeted for mutation by CIRSPR/Cas and caused delayed maturation (Yang et al. 2017). Another study showed that CRISPR/Cas9-engineered mutations on lncRNA1459 could delay the process of tomato fruit ripening (Li et al. 2018a).

Besides, the CRISPR/Cas genome editing system is widely applied to promote fruit quality. For example, the non-proteinogenic amino acid, γ-Aminobutyric acid (GABA), encoded by glutamate decarboxylase (GAD) gene *SlGAD2* and *SlGAD3*, is able to reduce blood pressure. CRISPR/Cas9 edited mutations in *SlGAD2* and *SlGAD3* resulted in enriched GABA in tomatoes (Nonaka et al. 2017). After that, five key genes in the GABA metabolism were edited by a multiplex pYLCRISPR/Cas9 system. Single to quadruple mutants with increased GABA contents were obtained (Li et al. 2018c). In addition, CRISPR/Cas constructs were applied to target *VACULAR PROCESSING ENZYME 5* (*SlVPE5*), a negative regulator of sugar accumulation in tomatoes, and *INVERTASE INHIBITOR HOMOLOG 1 (INVINH1*) that specifically inhibits cell wall invertase activity, leading to significantly increased sugar content. *SlINVINH1* and *SlVPE5* were demonstrated to be synergistic in repressing soluble sugar accumulation (Wang et al. 2021a). Ascorbate, also known as vitamin C, acts as antioxidant, which is beneficial to human health. A cis-acting uORF of GDP-L galactose phosphorylase (GGP) was edited by CRISPR/Cas9 system to obtain ascorbate-enriched fruits with impaired floral organ architecture (Deslous et al. 2021). Finally, CRISPR/Cas9 system has been applied to improve carotenoid content. The *STAY*-*GREEN* (*SGR*) gene encodes chloroplast-targeted proteins and is critical in plant chlorophyll II degradation (Park et al. 2007; Hortensteiner 2009). SlSGR1 directly interact with a key carotenoid synthetic enzyme, PHYTOENE SYNTHASE (PSY1). Silencing the *SGR1* gene in transgenic tomato fruits led to increased lycopene and β-carotene concentration at the mature green stage to red stage (Luo et al. 2013). Homologous chromosome recombination was induced via the CRISPR/Cas9 system, resulting in yellow tomato fruits in *psy1* mutants in contrast to red sectors in wild types (Filler Hayut et al. 2017).

##### Banana

As an important staple crop, banana is widely grown in more than 130 countries worldwide (Shao et al. 2020). Since banana is a polyploid and parthenocarpic fruit, the traditional asexual breeding method is less effective (Kaur et al. 2016). CRISPR/Cas-derived genome editing could be an effective approach to promote banana breeding. CRISPR/Cas9 was proven to be an effective tool in mediating *RAS*-*PDS* editing in the banana cultivar ‘Rasthali’ with a total editing efficiency of 59% (Kaur et al. 2018). Subsequently, a multiplexed approach via the polycistronic tRNA-gRNA system was applied to target *PDS*. A 100% mutation rate was reached among 19 regenerated plants, including triallelic deletions or insertions in banana ‘Williams’ (AAA genome subgroup). Two CRISPR/Cas9 vectors were tested in this system, one containing maize polyubiquitin promoter and the other containing CaMV 35S promoter to drive *SpCas9* gene expression. The result showed that only the maize *UBI1* promoter was effective in generating *PDS* mutations (Naim et al. 2018). However, a double CaMV35S promoter was shown to be effective in another system in targeting the same *PDS* gene. This CRISPR/Cas9 construct containing two PDS gRNAs was delivered into embryogenic cell suspension cultures of the banana cultivar, ‘Sukali Ndiizi’ (AAB genome subgroup), and plantain cultivar, ‘Gonja Manjaya’ (AAB genome subgroup). The mutation efficiency of both cultivars reached up to 100% among the 18 independent sequenced albino plants (Ntui et al. 2020). These studies provided a methodological framework for banana gene editing.

The CRISPR/Cas9 system has been applied to modulate plant architecture in banana. For example, dwarf banana varieties are suitable for mechanized plant maintenance and fruit harvest (Dash and Rai 2016). GA is one of the most important determinants of plant height (Sasaki et al. 2002), and the GA biosynthesis gene, *MaGA20ox2*, was edited by CRISPR/Cas9 in banana cultivar ‘Gros Michel’. Two sgRNAs of *MaGA20ox2* were driven by either *OsU6a* or *OsU3* promoter in pYLCRISPR/Cas9Pubi-H. Seven semi-dwarf mutant lines were obtained out of 152 independent transgenic lines (Shao et al. 2020).

CRISPR/Cas9 system has also been applied to improve banana fruit quality and extend its shelf life. β-carotene is an precursor of Vitamin A (Grune et al. 2010). Recently, two groups successfully increased β-carotene accumulation in banana through genome editing. In one study, the fifth exon of the *LYCOPENE EPSILON-CYCLASE* (*LCYε*) was targeted, and 10 of 12 *lcyε* mutant lines showed up to six-fold increased β-carotene content compared with the unedited plants (Kaur et al. 2020). In another study, the *CAROTENOID CLEAVAGE DIOXYGENASES* (*CCDs*) gene, which is a key enzyme modulating carotenoids degradation, was mutated by CRISPR/Cas9 and the mutation caused increased β-carotene accumulation (Awasthi et al. 2022). Both groups used the same plant-specific genome editing vector, pRGEB31, containing rice *snoRNA U3* promoter, and the acquired *CCD4* edited plants were transgene-free as they were generated by particle bombardment. Fruit shelf life greatly affects their economic value. By using the pYLCRISPR/Cas9Pubi-H CRISPR/Cas9 vector, *AMINOCYCLOPROPANE-1-CARBOXYLATE OXIDASE 1* (*MaACO1*) gene, expression of which converts 1-aminocyclopropane-1-carboxylic acid (ACC) into ethylene, was mutated which led to reduced ethylene production and increased shelf life without obvious vegetative growth defects (Hu et al. 2021).

##### Kiwifruit

Kiwifruit (*Actinidia chinensis*) has been called ‘the king of fruits’ due to its high vitamin C, minerals, and dietary fiber content (Huang et al. 2013). *Actinidia* are dioecious woody perennial species with long juvenility and crossing cycles, which has constrained genetic analysis and breeding. The precocious flowering habit and hermaphroditism are desired traits in kiwifruit breeding. Genome editing of kiwifruit has been reported using the conventional CRISPR/Cas9 system and polycistronic tRNA-sgRNA cassette (PTG)/Cas9 system. Phosphatidylethanolamine-binding protein (PEBP) genes are homologs to Arabidopsis *FLOWERING LOCUS T* (*FT*) and *TERMINAL FLOWER1* (*TFL1*), which are key regulators of flowering time. A Polycistronic tRNA-sgRNA cassette driven by the *Arabidopsis U6–26* promoter or *U3* promoter was used to target PEBP family genes *CENTRORADIALIS* (*CEN*) and *CENTRORADIALIS 4* (*CEN4*), and the resulting female kiwifruit plants showed precocious terminal flowers and fruit development (Varkonyi-Gasic et al. 2019). Similarly, the male-specific *SHY GIRL* (*SyGl*) gene, a homology to type-C cytokinin response regulators, has a role in repressing gynoecium development as well as feminization. In the male kiwifruit cultivar ‘Bruce’, *CEN4*/*SyGl* knockout lines generated by CRISPR/Cas9 showed rapid-flowering hermaphrodites (Varkonyi-Gasic et al. 2021). Another group also reported two tetraploid male kiwifruit accessions with mutations in the *SHY GIRL* (*SyGI*) gene. A CRISPR/Cas9 multiplexing system containing RNA endoribonuclease *Csy4* was employed, and the nucleotide modification ratio ranged from 99.76–99.85% at the target sites (De Mori et al. 2020). The PTG/Cas9 system has been applied to regulate other genes in flower development in kiwifruits. For example, *BROTHER OF FT AND TFL1* (*BFT*) gene which belongs to the subclade of CEN/TFL1 lineage in the PEBP family and is involved in axillary inflorescence development. Four sgRNA were designed to target *AcBFT2* and *AcBFT3*, and the resulting *Acbft* mutant lines showed an overgrowing phenotype, delayed dormancy, and early budbreak (Herath et al. 2022). Besides, the kiwifruit *FLOWERING LOCUS C-LIKE* (*AcFLCL*) gene without the specific C-terminal signature sequences was edited by the multiplex tRNA-gRNA system, and the resulting edited lines showed delayed flower budbreak. The discrepancy in *FLC-like* gene function between kiwifruit and other model plants may be due to the mis-annotation of MADS-box genes (Voogd et al. 2022). These efforts provide an important genetic locus for kiwifruit breeding.

### Plant immunity

#### Biotic stresses

##### Bacterial pathogens

The CRISPR/Cas9 system has been successfully applied in citrus resistance to biotic stresses. Citrus canker, caused by bacterium *Xanthomonas axonopodis*, is a major disease of citrus and resuls in substantial yield loss globally (Zou et al. 2021). *Citrus sinensis LATERAL ORGAN BOUNDARY 1* (*CsLOB1*) belongs to the LATERAL ORGAN BOUNDARY DOMAIN (LBD) gene family and functions as a disease susceptibility gene in citrus canker. CRISPR/Cas9 editing in the epicotyl explants of Duncan grapefruit (*Citrus paradise*) led to six transgenic lines exhibiting various degrees of resistance to the citrus canker (Jia et al. 2017). Another study showed that editing the *CsLOB1* promoter resulted in homozygous plants with enhanced resistance to citrus canker (Peng et al. 2017). CRISPR/Cas12a (Cpf1) belongs to the Class II type V CRISPR system. Cas12a is more likely to cut and edit the target site effectively, resulting in larger deletions than those induced by Cas9 (Zetsche et al. 2015; Tang et al. 2017) or enhanced HDR activity (Wolter and Puchta 2019; Li et al. 2020b). CRISPR/Cas12a was subsequently applied in Duncan grapefruit to edit *CsPDS* and the promoter of *CsLOB1*. In this study, LbCas12a was driven by either 35S or Yao promoter, and the 35S promoter showed higher editing efficiency (Jia et al. 2019). Recently, the ABE system was applied in grapefruit and sweet orange to edit the promoter of *LOB1*, resulting in increased resistance to canker pathogen *Xcc* (Huang et al. 2022). In addition, *Xcc* has been demonstrated to induce the *WRKY* transcription factor *CsWRKY22*. CRISPR/Cas9 mediated knockout of *CsWRKY22* led to increased resistance of Wanjincheng orange (*Citrus sinensis* Osbeck) to *Xcc* (Wang et al. 2019b).

In addition, *Xanthomonas* could also cause substantial impacts on banana and tomato. Banana Xanthomonas Wilt (BXW) disease, caused by *Xanthomonas campestris* pv. *Musacearum*, can result in up to 100% yield losses, especially in the brewing type banana. CRISPR/Cas9-based genome editing of the disease-causing susceptibility (S) genes has been reported to increase resistance to BXW. For example, S gene *DOWNY MILDEW RESISTANCE 6* (*DMR6*) was edited by CRISPR/Cas9 and the *Musadmr6* mutants showed increased resistance to BXW without any growth penalty (Tripathi et al. 2021). In tomatoes, *Sldmr6–1* was shown to be more resistant to different pathogens, including *Xanthomonas* spp. (Thomazella et al. 2021). In total, these susceptibility genes and negative regulators of disease resistance might be good targets for CRISPR/Cas to enhance plant resistance (Tripathi et al. 2020).

Furthermore, fire blight is caused by the bacterium *Erwinia amylovora* and is a devastating disease affecting apple production. The interaction between the *E. amyloyora* effector, DspA/E, and the kinase domain of DspA/E-INTERACTING PROTEINS OF *Malus domestica* (MdDIPM) was found to be responsible for the pathogenesis (Meng et al. 2006). The *DIPM-1*, *DIPM-2*, and *DIPM-4* in the apple cultivar *Golden delicious* fruit was edited through direct delivery of purified CRISPR/Cas9 ribonucleoproteins (RNPs) to increase resistance of apple to fire blight disease. In this system, a heat-shock inducible promoter was used to drive Cas9 expression, and the editing efficiency ranged from 1.4–29% (Malnoy et al. 2016).

##### Fungal pathogens

The CRISPR/Cas9 system has been widely used in fruit crops to increase plant immune responses against fungal diseases. *Botrytis cinerea* (*B. cinerea*) is one of the most notorious necrotrophic fungal pathogens infecting more than 1000 host plants (Xiong et al. 2018). To increase resistance to *B. cinerea*, the grape transcription factor *VvWRKY52* was edited using *Agrobacterium* mediated CRISPR/Cas9 system. *Vvwrky52* mutantlines exhibited increased resistance to *B. cinerea* (Wang et al. 2018a). In addition, It was demonstrated that *SlMYC2* plays an essential role in tomato growth and disease resistance against *B. cinerea.* The *myc2* knockout mutants showed decreased resistance against *B. cinerea*, accompanied by a greater number of flowers and decreased fruit set compared to the wild type (Shu et al. 2020).

Powdery mildew (PM) is a destructive fungal disease caused by the fungal pathogen *Erysiphe necator*, and is responsible for considerable yield losses in fruit crops. *MILDEW RESISTANCE LOCUS O* (*MLO*) family genes function as susceptible factors for powdery mildew infection. Through direct delivery of purified CRISPR/Cas9 ribonucleoproteins into protoplasts, MLO-7 was targeted for mutagenesis which caused significantly increased resistance to powdery mildew (Malnoy et al. 2016). This method has subsequently been improved to obtain whole transgenic plants (Osakabe et al. 2018). Later, tomato *SlMLO1* was knocked out using the CRISPR/Cas9 system, leading to increased resistance to powdery mildew in *slmlo1* mutant lines (Pramanik et al. 2021). In grapes, *vvmlo3* edited lines generated by CRISPR/Cas9 also exhibited increased resistance to powdery mildew (Wan et al. 2020). These studies demonstrated the potential to achieve high PM resistance in fruit crops by simply knocking out the *MLO* locus.

Fusarium wilt, caused by *Fusarium oxysporum* f.sp. *niveum* (*FON*), is lethal to plants and severely affects global watermelon production. *FON* progresses along xylem vessels leading to hollow and dried-out stems (Zhang et al. 2015a). Recently, a CRISPR/Cas9 system was also used to knock out the *PHYTOSULFOKINE1* (*ClPSK1*) gene encoding the phytosulfokine precursor, resulting in increased watermelon resistance to *FON* (Zhang et al. 2020b).

CRISPR/Cas has also been used to increase plant resistance to oomycete. The pathogenesis-related 4 (PR4) protein has critical roles in resisting to this disease, as the *VvPR4b* knockout lines generated by CRISPR/Cas9 show increased susceptibility to the oomycete pathogen *Plasmopara viticola* (Li et al. 2020a). In addition, CRISPR/Cas was used to target small RNA to increase plant resistance to oomycete. For instance, using CRISPR/Cas9 system targeting micro-RNA482b and micro-RNA482c simultaneously would significantly increase mutant plant resistance against *Phytophthora infestans* (Hong et al. 2021).

##### Virus

The CRISPR/Cas9 system has also been widely applied to increase resistance against viruses and was first used in cucumber to edit the elF4E (eukaryotic translation initiation factor 4E) gene, causing broad viral resistance in transgene-free T3 progeny (Chandrasekaran et al. 2016). In addition, *TOBAMOVIRUS MULTIPLICATION1* (*TOM1*) gene has been reported to confer resistance against the tomato brown rugose fruit virus (ToBRFV). Recently, all four tomato homologs of *TOM1* were mutated by the CRISPR/Cas9 system, and the quadruple-mutant showed increased resistance to ToBRFV without obvious defects in growth or fruit production (Ishikawa et al. 2022).

#### Abiotic stresses

CRISPR/Cas9 has also been applied to increase resistance to abiotic stresses. For instance, mutagenesis of the cyclic nucleotide-gated ion channels (CNGCs) homologous gene *MdCNGC2* was achieved using a CRISPR/Cas9 toolkit containing pHDE-35S-Cas9-mCherry-UBQ and *MdU6* promoter-driven sgRNA, leading to constitutive accumulation of SA and increased expression of several defense-related genes in apple calli (Zhou et al. 2020a).

In addition, the CRISPR/Cas system has been applied to increase resistance to abiotic stress, such as iron (Fe) deficiency, cold stress, and herbicide resistance. Recently, tomato *SQUAMOSA PROMOTER-BINDING PROTEIN-LIKE* (*SPL*) transcription factor (*SlSPL*-*CNR*) knockout lines generated using CRISPR/Cas9 showed intensified Fe deficiency responses (Zhu et al. 2022), suggesting that *SlSPL-CNR* plays a negative role in regulating Fe-deficiency response in tomato. The cold-responsive *C-REPEAT BINDING FACTORS 1* (*SlCBF1*) was edited using the CRISPR/Cas9 system to investigate the mechanism of chilling response in tomatoes, and the resulting *slcbf1* mutants show severe chilling phenotype (Li et al. 2018b). Furthermore, mutations of *PHYTOCHROME A SIGNAL TRANSDUCTION 1 (VaPAT1*) obtained by genome editing showed reduced accumulation of JA during cold stress responses (Wang et al. 2021d). Finally, herbicide resistance in crops is in great need for plant breeding. 5-Enolpyruvylshikimate-3-phosphate synthase (EPSPS) is a toolkit enzyme in the biosynthesis of aromatic amino acids such as tryptophan, tyrosine, and phenylalanine (Yi et al. 2016; Leino et al. 2021). *ACETOLACTATE SYNTHASE* (*ALS*) is the key enzyme gene for the biosynthesis of branched-chain amino acids, valine, leucine, and isoleucine (Yu and Powles 2014). The CRISPR/Cas-derived CBE system has been applied to edit the *ALS* gene in watermelon and apple, leading to transgene-free homozygous mutant plants and significantly enhanced chlorsulfuron resistance (Tian et al. 2018; Malabarba et al. 2020). A recent study showed that knockout mutants of *SlALS* and *SlEPSPS* generated by CRISPR/Cas9 both resulted in increased herbicide resistance (Yang et al. 2022).

## Challenges and improvements of applying genome editing in fruit crops

Although genome editing has been developed and applied in plant development and plant immunity improvement over the past 10 years, many challenges and problems still need to be overcome to improve genome editing systems. Generally, six major obstacles hinder the application of genome editing in fruit crops: 1) It is challenging to predict high-efficient sgRNAs; 2) the protein expression and enzyme activity of Cas protein derivates are not fully optimized; 3) the efficiency of sgRNA and Cas delivery system is still low; 4) the specificity of genome editing in plants needs further improvement; 5) the regenerate process of the gene-edited plantlets is time-consuming and has low efficiency; 6) transgene-free genome editing is another major challenge, especially for these polyploid fruit crops.

### Improving genome editing efficiency by optimizing the expression of sgRNA and Cas proteins

Various strategies have been developed to optimize the CRISPR components to increase the ratio of successful gene editing in plants. The results show that GC contents of sgRNA close to 65% will likely achieve the highest editing efficiency (Ren et al. 2014). Thus, a selection of sgRNA with high GC content and increased *Streptococcus pyogenes* Cas9 (*SpCas9)* expression was adopted to achieve high-efficient genome editing (Ren et al. 2019). The expression of sgRNA was driven by the RNA polymerase III (Pol III) promoter of small nuclear RNA (snRNA) genes, such as *U3* and *U6* (Lowder et al. 2015). Generally, *AtU6* and *AtU3* promoters have been demonstrated to be functional in many dicot plants, including strawberry, grape, and watermelon. However, there is increasing evidence that applying the native U6 promoters increases CRISPR/Cas9 editing efficiency in cereal crops such as cotton (Long et al. 2018) and soybean (Sun et al. 2015). Recently, the method has also been expanded to fruit crops. For instance, a strawberry native U6–2 promoter was used to drive sgRNA and led to high-efficient genome editing in woodland strawberries (Zhou et al. 2018). A banana codon-optimized Cas9 and a *MaU6c* promoter were used and caused an increased mutation efficiency by four folds (Zhang et al. 2022). The grape *VvU3*, *VvU6*, and *VvUBQ2* promoters have been employed to drive the expression of sgRNA and Cas9, respectively, resulting in an improved genome editing efficiency ranging from 14.65 to 22.10% (Ren et al. 2021). However, in many non-model plants, Pol III promoters have not been characterized. In contrast to sgRNA, the Cas9 protein expression is usually driven by the RNA polymerase II (Pol II) promoter (Lowder et al. 2015). Coordinated expression of Cas9 and the sgRNA is usually challenging. Therefore, a system in which both Cas9 and sgRNA were driven by the same Pol II promoter has been developed recently. In this system, Cas9 and the sgRNA(s) were separated by ribozyme cleavage sites and were driven by a single Pol II. The result shows this system could achieve an editing efficiency of up to 30% at *OsPDS* target sites in rice, and could target multiple genes simultaneously (Tang et al. 2016). Nevertheless, it is still a challenge to apply the combined expression method of sgRNA and Cas9 in horticultural plants.

In addition, genome editing efficiency was also determined by Cas proteins. Among class 2 CRISPR/Cas systems, type II Cas9, type V-A Cas12a (Cpf1), and type V-B Cas12b are all RNA-guided endonucleases (Makarova et al. 2020). Cas9 and Cas12b require sgRNAs, while Cas12a need only a crRNA to achieve precise genome editing (Makarova et al. 2020). Cas9 recognizes G-rich protospacer adjacent motifs (PAMs), whereas Cas12a/b recognize T-rich, and therefore extends the usage scope within the genome (Ming et al. 2020). CRISPR/Cas12a has been successfully used in plants, Drosophila and zebrafish (Endo et al. 2016; Port and Bullock 2016; Moreno-Mateos et al. 2017). It contains three different forms, including *Acidaminococcus* sp. BV3L6 Cas12a (AsCas12a), *Francisella novicida* Cas12a (FnCas12a), and *Lachnospiraceae bacterium ND2006* Cas12a (LbCas12a). LbCas12a has been reported to perform better than AsCas12a in rice (Tang et al. 2017). Moreover, LbCas12a has been applied in horticultural plants such as citrus (Jia et al. 2019), apple (Schropfer and Flachowsky 2021), and banana (Wu et al. 2020) to edit genes involved in resistance to citrus canker or albino phenotype. Recently, CRISPR/Cas12a-based nucleic acid detection platform was developed and optimized to diagnose RNA viruses in apples with high specificity and sensitivity (Jiao et al. 2021).

Multiplex genome editing could be achieved by stacking multiple sgRNA-expressing cassettes in one plasmid or by using multiple constructs. However, it is challenging due to the limitation of the delivery method and plasmid vector capacity (Xie et al. 2015). Therefore, the PTG/Csy-type (CRISPR system yersinia) ribonuclease 4 (Csy4)-gRNA system has been well developed in CRISPR/Cas9 to edit multiple genes simultaneously and overcome gene redundancy by designing multiple gRNAs in a tandem array with linker sequence recognized by endoribonuclease Csy4. It is reported that the PTG and Csy4 mediated multiplex gene editing has been applied in tomato (*Solanum lycopersicum*), tobacco (*Nicotiana tabacum*), wheat (*Triticum aestivum*), barley (*Hordeum vulgare*), *Medicago truncatula* (Cermak et al. 2017), and kiwifruit (Wang et al. 2018b). The PTG/Cas9 system exhibited a 10-fold higher editing efficiency than the conventional CRISPR/Cas9 system (Wang et al. 2018b). Recently, an optimized system was developed in which one Pol II promoter can drive up to six tRNA-gRNA2.0 cassettes. The multiplexed tRNA-gRNA2.0 shows many advantages for multiplexed gene activation than conventional multiplex U3-gRNA2.0 (Pan et al. 2021b).

### Improving the delivery efficiency of CRISPR/Cas reagents

Effective application of CRISPR/Cas9 in plants requires a robust and effective method to deliver CRISPR/Cas reagents into plant cells (Zhu et al. 2020). Currently, the conventional delivery methods are still *Agrobacterium*-mediated transformation and particle bombardment (Ozyigit and Yucebilgili Kurtoglu 2020), although a considerable number of new methods have emerged in recent years, including polyethylene glycol (PEG)-mediated protoplast transfection combined with RNP (Shen et al. 2014; Woo et al. 2015), viral vectors-mediated delivery system (Ellison et al. 2020), and nanoparticles (Demirer et al. 2019; Gao 2021). Among these methods, RNP-mediated genome editing with protoplasts has proven to be a promising strategy for transgene-free genome editing, as it has been successfully applied in apples (Malnoy et al. 2016), grapes, bananas (Wu et al. 2020), cabbages (Murovec et al. 2018) and wheat (Liang et al. 2017). PEG-mediated banana protoplast transformation was developed with pUBI-Cas9 and LbCas12a. By targeting the *PDS* gene, this system showed an editing efficiency that ranged from 0.15–1.04% (Wu et al. 2020). Nevertheless, the protoplast isolation and protoplast regeneration process are extremely hard for many plants, preventing the application of this method in achieving transgene-free genome editing in many fruit crops. Viral vector systems refer to viral vector-mediated delivery of sgRNA into Cas9 overexpressing plants. It is simple and does not need to integrate into plant genome DNA (Laforest and Nadakuduti 2022). Nanoparticle is a novel method with enhanced efficiency of DNA entry into plants that are recalcitrant to *Agrobacterium*-mediated transformation (Wang et al. 2022b). Nanoparticle-based transformation, which are of various types, has also been used to generate transgenic plants without tissue culture regeneration in monocots and dicotyledons (Lv et al. 2020).

Recently, a newly developed tissue culture-free system named cut-dip-budding delivery was developed, in which transformed buds were grown directly from transgenic hairy roots because of the root-suckering ability. This system has been applied in herbaceous and woody plants (Cao et al. 2023) and enables efficient transformation and gene editing with a simple system and may be applied to a wide range of plant species.

### Improving the specificity of genome editing

Off-target effects are one of the major concerns in genome editing. Several strategies have been developed to enhance the specificity of CRISPR/Cas. One direct method is designing high-efficient gRNAs (Bae et al. 2014). It has been demonstrated that sgRNA sequence with GC contents between 40 and 60% results in the highest on-target activity (Ren et al. 2014). The structure of gRNA also affects the chances of off-target editing (Naeem et al. 2020). Chemical modification of RNA, such as integrating bridged and locked nucleic acid in the guide sequence to form a dynamic RNA-DNA duplex, has been reported to improve specificity by about 25,000 folds (Cromwell et al. 2018). In addition, the specificity of CRISPR/Cas can be improved by transiently expressing the pre-assembled Cas9-gRNA ribonucleoproteins as reported in maize (Svitashev et al. 2016) and wheat (Liang et al. 2017). Other ways to reduce off-target effects include using engineered precision variants of Cas9, Cas12a, and deaminases (Zhang et al. 2019; Jin et al. 2020a) or high-fidelity Cas9, such as the enhanced specificity SpCas9 (eSpCas9) (Slaymaker et al. 2016; Chen et al. 2017). However, more efforts are needed to improve the specificity of existing genome editing tools or create new editors with higher specificity.

### Optimizing transformation and regeneration systems

Current regeneration mainly relies on tissue culture, which is time-consuming and laborious. Low regeneration rates have been the bottleneck of genetic transformation in many plant species. It usually takes a long time and leads to chimeras during the period of tissue culture. Taking the apple as an example, it is well-known that apple transformation is time-consuming and usually takes more than 6 months, and the generation of genome-edited apple trees takes several years. Besides, over 85% of edited *MdPDS* lines were chimeric (Nishitani et al. 2016; Charrier et al. 2019). To tackle this problem, researchers reported an adventitious regeneration protocol by adding an adventitious regeneration step from the leaves of primary transgenic plants, which led to reduced chimerism in apples and pears (Malabarba et al. 2020). In addition, the rate of chimeras could be decreased by specifically expressing CRISPR cassettes in germline cells for heritable genome modification. Germ-line-specific Cas9 system (GSC) has been demonstrated to reduce the chimeras rate in which male gametocyte-specific promoters or egg cell-specific promoters were applied (Wang et al. 2015b; Mao et al. 2016).

Recently, an intrinsic growth-regulating factor was used to promote the efficiencies of plant regeneration and genome editing (Omidbakhshfard et al. 2015). Nowadays, it has been demonstrated that *GROWTH-REGULATING FACTORs* (*GRFs*), *GRF-INTERACTING FACTORs* (*GIFs*), and *GRF-GIF* chimeric genes can improve regeneration efficiency dramatically in various monocot and dicot plants, including watermelon (Feng et al. 2021b; Pan et al. 2022), wheat, triticale, rice (Debernardi et al. 2020), and citrus (Kong et al. 2020). Two groups successfully applied developmental regulators, *GRF-GIF* chimeras, to improve watermelon transformation efficiency by 20% without any obvious negative effects on plant growth (Feng et al. 2021b; Pan et al. 2022). In addition, the expression of *WUSCHEL* (*WUS*) and *BABY BOOM* (*BBM*) increased regeneration efficiency (Lowe et al. 2016). Nevertheless, *GRFs-GIFs* chimeric genes are more advantageous than *WUS* and *BBM*, as they have no apparent side effects during the regeneration process (Gao 2021). These studies have greatly accelerated the application of genome editing in modern breeding in fruit crops.

### Obtaining the transgene-free plants

The commercialization of genome-edited plants has been a public concern because it is difficult to remove transgenes via traditional crossing and segregation in fruit crops due to its long juvenile period (Prado et al. 2014). Transgene-free genome editing provides a promising alternative to conventional crop breeding and enables modern breeders to pursue rapid and efficient germplasm innovation in the field (Zhou et al. 2020b). However, the efficiency of obtaining transgene-free plants has always been low. Strategies to move out exogenous DNA include lipid transfection, viral vectors, and delivery of components directly as functional sgRNA and Cas9 protein. For example, one cationic lipid transfection agent, lipofectamine, was reported to transfect the Cas9 construct containing a gRNA targeting the citrus *NONEXPRESSOR OF PATHOGENESIS-RELATED 3* (*CsNPR3*) gene into citrus protoplasts. Using lipofectamine with PEG significantly improved transfection efficiency by up to 51% (Mahmoud et al. 2022). In other reports, RNA viruses could assist gene modification without integrating into the plant genome. For example, the tobacco rattle virus (TRV) has been employed to deliver sgRNA in dicots (Ali et al. 2015; Cody et al. 2017). In addition, Sonchus yellow net rhabdovirus (SYNV) has been engineered to carry Cas9 and sgRNA for DNA-free editing in plants (Ma et al. 2020). Finally, the direct introduction of an RNP complex consisting of gRNA and Cas9 into host cells, combined with a high-efficient protoplast regeneration protocol, might be a feasible way to overcome this barrier (Osakabe et al. 2018). This method has been reported to be successful in many plants, including Arabidopsis, tobacco, lettuce, rice (Woo et al. 2015), grape, apple (Kanchiswamy 2016; Malnoy et al. 2016), and tomato (Lin et al. 2022a). For fruit crops, CRISPR/Cas9 RNP was delivered into grapevine and apple protoplasts to induce transgene-free genome-edited plants (Malnoy et al. 2016). This RNP method was improved according to a stepwise protocol for designing and transferring CRISPR/Cas9 components into the apple and grapevine protoplasts, followed by highly efficient verification and regeneration methods. RNP based transgene-free genome editing system has also been developed to accelerate tomato breeding. Relying on an optimized protoplast regeneration protocol of *Solanum peruvianum*, A DNA-free CRISPR/Cas9 genome editing system has been established. With this system, the RNA-DEPENDENT RNA POLYMERASE 6 (*SpRDR6*), SUPPRESSOR OF GENE SILENCING 3 (*SpSGS3*), PATHOGENESIS-RELATED PROTEIN-1 (*SpPR-1*), PROSYSTEMIN (*SpProSys*), and MILDEW RESISTANT LOCUS O (*SpMLO1*) genes involved in small interfering RNAs biogenesis or fungal resistance, were mutated in both diploid and tetraploid regenerants (Lin et al. 2022a). The RNP approach allows transgene-free genome editing that takes as short as 2–3 weeks to regenerate plants, while plasmid mediated procedure usually takes over 3 months (Osakabe et al. 2018).

Another system, a heat-shock inducible FLP/FRT recombination system, has been specifically designed to remove the T-DNA harboring the expression cassettes for CRISPR/Cas9. With the CRISPR/Cas9-FLP/FRT gene editing system, *MdDIPM4*-edited apple plants carrying a minimal trace of exogenous DNA were obtained (Pompili et al. 2020). A similar system based on designing and recognizing two additional target sites next to the LB and RB sites could lead to the complete removal of T-DNA and transgene-free editing (Dalla Costa et al. 2020). Obtaining transgene-free plants has always been a major goal for the application of genome editing in crop breeding, especially for these perennials and polyploid fruit crops.

## Future directions and remarks

### Application of novel techniques in fruit crops

The widely used genome editing technologies, such as CRISPR/Cas system, were effective in causing mutations and generating loss-of-function mutants. However, in many cases, the overexpression of genes to get beneficial agronomic traits is more attractive for plant breeders. Conventional gene overexpression system usually requires the introduction of exogenous transgene and cause many biological and ethical issues. Therefore, it is necessary to develop novel techniques to activate gene expression by introducing exogenous transgene components. A newly developed system with chemically modified donor DNA and CRISPR/Cas has been reported to insert sequences of up to 2049 base pairs. In this system, a 5′-phosphorylated double-stranded oligodeoxynucleotide (dsODN) was designed to stabilize donor DNA and CRISPR/Cas system. Translational enhancers could be inserted into the 5′ untranslated region of the target gene, increasing target gene expression (Lu et al. 2020).

Precise genome editing, including targeted base substitutions, gene insertions/deletions, and gene replacements, offer advanced ways to knockout, modify, or alter gene activity; hence, it is more versatile than the standard gene knockout (Gao 2021). Base editor is a powerful precision tool that can generate programmable single DNA base changes. However, the substitutions in the target gene are currently limited to C•G to T•A and A•T to G•C, which greatly impeded the application of the base editor system in plants (Gao 2021; Kurt et al. 2021; Zhao et al. 2021). Surprisingly, a transversion base editor has already been reported to cause C to G base edits (CGBEs) (Sretenovic et al. 2021). Nowadays, base editing has been successfully applied in many cereal crops, including rice and maize (Lin et al. 2020; Zhu et al. 2020), as well as in many fruit crops, including strawberry (Xing et al. 2020), orange (Huang et al. 2022), apple (Malabarba et al. 2020), and tomato (Shimatani et al. 2017). However, the development and application of broad-spectrum base editor tools still have a long way to go.

Compared to the CRISPR/Cas system and base editor, the potential of prime editor in fruit crop improvement is quite exciting, as it has the advantage of inserting predesigned large DNA fragments. However, the application of prime editor in horticultural plants is still rare. The bottleneck for its application resides in its extremely low efficiency and difficulty in assembling. To improve the efficiency of genome editing, a series of strategies have been developed. For example, it has been reported that the efficiency of prime editing could be increased by inhibiting specific DNA mismatch repair (MMR) proteins (Chen et al. 2021). Besides, pegRNA has been optimized by incorporating structured RNA motifs to improve prime editing efficiency without increasing off-target (Nelson et al. 2022). In addition, a pair of pegRNAs targeting both DNA strands could increase genome editing efficiency by about 2.7-fold more than the traditional prime editor system with only one DNA strand edited (Zhuang et al. 2022). In another report, the twin prime editing (twinPE) system, which contains a prime editor protein and two pegRNAs, has been employed to fulfill a targeted integration of large-size DNA fragments (Anzalone et al. 2022). Therefore, prime editors hold great potential to achieve long DNA fragment insertion and transgene-free over-expression that could be the most attractive point for the further development and application of prime editing systems in fruit crop breeding.

The ultimate goal of genome editing-mediated plant breeding is to harvest plants with combined beneficial traits and eliminate unfavorable traits. However, genes coding for beneficial traits is often linked to traits with detrimental features. It has been reported chromosome restructuring could overcome this shortage of linkages between traits. With egg-cell-specific Cas9 nuclease from *Staphylococcus aureus*, a directed chromosome modification of reciprocal translocations in the Mbp range between heterologous chromosomes could be induced (Beying et al. 2020). Novel strategies, including chromosome engineering, hold great potential in genome-edited plant breeding.

### Application of novel CRISPR/Cas-derived transcriptional activation/ repression and epigenetic regulation platforms

Gene function investigation mainly relies on conventional reverse genetics approaches, which lack the flexibility and scalability to modulate multiple genes simultaneously or precisely modify genes at epigenetic levels. CRISPR/Cas has made significant progress in generating desired edits at target sites. Beyond gene editing, CRISPR/Cas systems have great potential in modulating genes as a robust programmable platform. For instance, catalytically inactivated Cas variants (dCas) fused to diverse enzymes have expanded the scope of the CRISPR/dCas system, including CRISPR-mediated gene activation (CRISPRa), interference (CRISPRi), and epigenetic modifications. Engineered dCas9, dCas12a, and dCas12b fused to different enzymes could function as effective gene modulation platforms (Pan et al. 2021a).

CRISPR/dCas-mediated transcriptional activation has been developed from the first-generation system, including dCas9-VP64, dCas9-TV, and dCas9-SunTag, to the third-generation system, including dCas12a-TV and dCas12b-TV-MS2-VPR (Pan et al. 2021a). CRISPRa and CRISPRi systems have been improved by using various transcription effectors, RNA aptamers, and multimeric peptide arrays that can be incorporated to stimulate the recruitment of specific transcription effectors (Xu and Qi 2019). CRISPR/dCas-mediated gene expression modulation has been extensively used in model plants such as Arabidopsis (Ming et al. 2020) and rice (Tang et al. 2017; Gong et al. 2020). In grape, dCas9 was fused to transcriptional activators, six copies of TALEs and two copies of VP64, to get the dCas9-TV system. With this system, *CBF4* was targeted and increased gene expression and cold resistance (Ren et al. 2022). It shows its potential to expand this technology in other horticultural plants.

Epigenetic modifications, such as histone modification and DNA methylation, could modulate gene expression and contribute to plant breeding improvements. DECREASE IN DNA METHYLATION 1a (DDM1a) and DDM1b redundantly regulate DNA methylation level. A *Slddm1a Slddm1b* double mutant was created with CRISPR/Cas9, which exhibited reduced DNA methylation and pleiotropic phenotypes (Corem et al. 2018). CRISPR/dCas9-based epigenome editors could especially modulate the epigenetic level of target genes. This system has provided an unprecedented opportunity to investigate the relationship between epigenetic regulation and agronomically important biological processes. However, non-specific epigenetic changes are still the major concerns for applying CRISPR/dCas-mediated epigenetic regulation in plants.

**Fig. 5:**
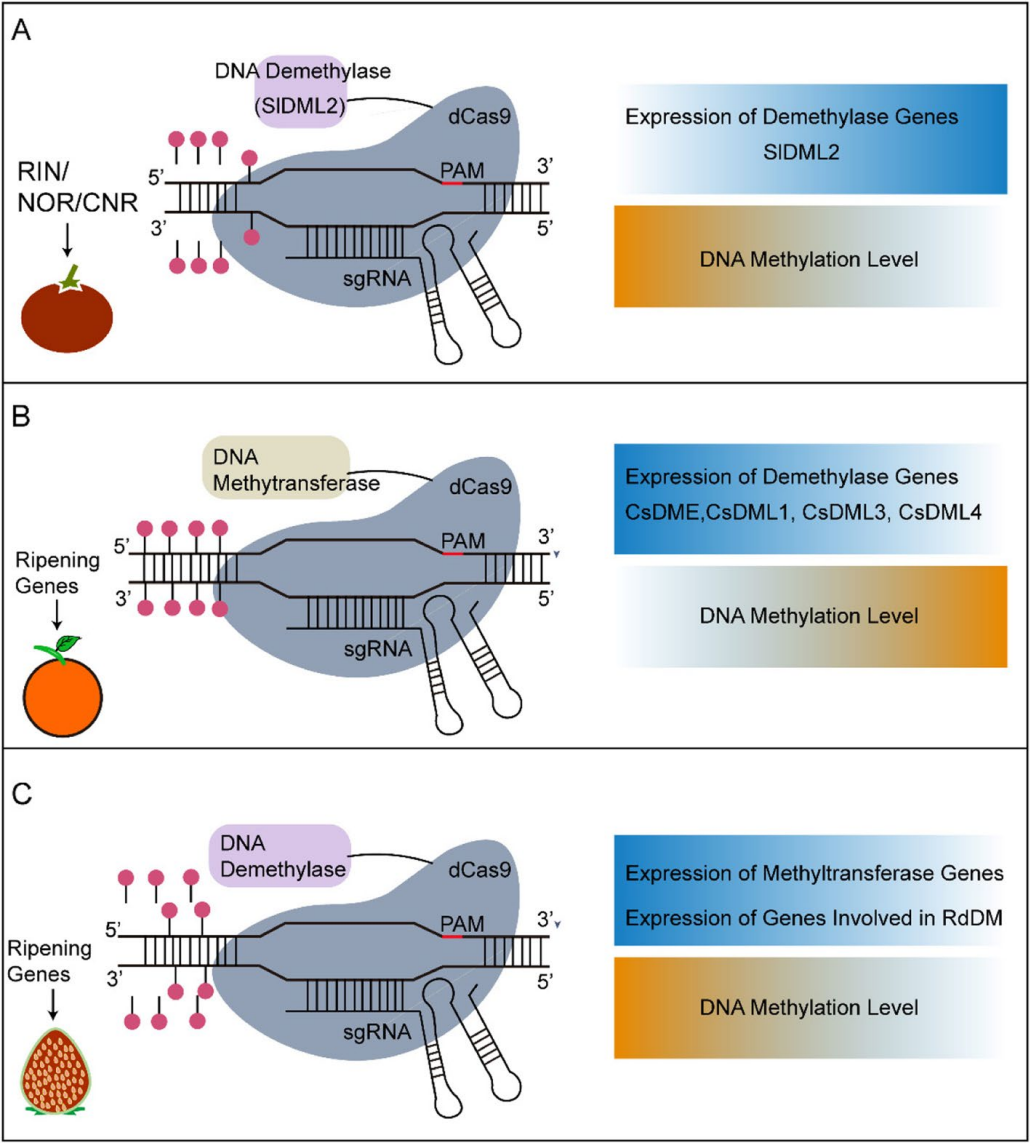
CRISPR/dCas9-based epigenetic modifier during fruit ripening process in tomato, sweet orange, and strawberry. **A** CRISPR/dCas9 based epigenetic modifier in tomato fruit ripening. The DNA demethylase can be engineered to fuse to dCas9 system. In tomato fruit ripening process, the expression of DNA demethylase *DME-LIKE 2* (*DML2*) increases, leading to decrease of 5-methlcytosine (5mC) DNA level at various gene loci, such as RIN, CNR, and NOR. **B** CRISPR/dCas9 based epigenetic modifier in sweet orange fruit ripening process. DNA methyltransferase could be engineered to fuse to dCas9 system. During sweet orange fruit ripening, DNA demethylase genes are downregulated, leading to the upregulation of 5mC DNA methylation level. **C** CRISPR/dCas9 based epigenetic modifier in strawberry fruit ripening process. DNA methyltransferase could be engineered to fuse to dCas9 system for promoting strawberry fruit ripening. As strawberry fruit ripening, the activity of RNA-directed DNA methylation is reduced, resulting in a decrease in the DNA methylation level. Diagrams were drawn based on publications (Liu et al. 2015; Cheng et al. 2018; Huang et al. 2019; Chen et al. 2020)

Emerging evidence suggests that DNA methylation plays an important and distinct role in fleshy fruit ripening. Tomato, a typical climacteric fruit, and strawberry, a typical non-climacteric fruit, undergo loss of global DNA methylation during fruit ripening. However, the mechanisms underlying DNA hypomethylation during the ripening of tomatoes and strawberries are different. Unlike tomatoes, in which the increased DNA demethylase gene *DML2* expression causes decreased 5mC levels during tomato fruit ripening (Lang et al. 2017), downregulation of RNA-directed DNA methylation (*RdDM*) is mainly responsible for the decreased 5mC levels during strawberry fruit ripening (Cheng et al. 2018). A more intricate example is the sweet orange. It was found the 5mC DNA methylation level is significantly increased during sweet orange fruit ripening, which is mainly caused by the downregulation of DNA demethylation genes (Fig. 5) (Huang et al. 2019). Thus, the overall DNA methylation level is not necessarily correlated with the ripening process in different fruit crops. Instead, the specific ripening-related genes involved in DNA methylation might be the main reasons. Therefore, further application of CRISPR/dCas9-based epigenome editing on specific fruit ripening genes may illustrate the roles of epigenetic regulation in different ripening types and in important biological traits in plants (Fig. 5). Such work is already being reported. For example, an epigenome editor named CRISPRoff has been developed, in which dCas9 was fused to functional domains of DNA methyltransferase to initiate DNA methylation to repress gene expression (Nunez et al. 2021). In addition, RNA epigenetic engineering tools targeting RNA epigenetic modifications have been developed (Liu et al. 2019; Wilson et al. 2020). These new tools will definitely facilitate basic research in fruit crops.

### Polyploid fruit crop breeding with high-efficient genome editing strategy

Traditional fruit crop breeding is time-consuming due to the genome complexity and long lifespan. In addition, high heterozygosity and polyploidy make it harder to apply genome editing in fruit crops. The application of the conventional CRISPR/Cas9 system in polyploid species (potato, oilseed rape, cotton, bread wheat, and strawberry) is challenging mainly due to two factors: 1) The relatively low efficiency of genome editing system makes it hard to edit all homologous alleles; 2) The transgene components are not easily removed from genome-edited lines (Weeks 2017; Martin-Pizarro et al. 2019).

To overcome these barriers, a doubled haploid (DH) technology has attracted more attention nowadays. DH accelerates the breeding process by achieving homozygosity in two generations, which is much shorter than the traditional cross methods (six to eight generations). Therefore, the production of haploids is urgently needed for the application of this technology in plant breeding. With haploid induction editing (HI-Edit) and haploid inducer-mediated genome editing (IMGE), target genome sequences of any elite commercial background can be modified by haploid inducer line combined with the CRISPR/Cas9 system (Kelliher et al. 2019; Wang et al. 2019a; Gao 2021). For example, *MATRILINEAL* (*MTL*) is a plant gene encoding sperm-cell-specific phospholipase. The *mtl* knockout mutants generated by CRISPR/Cas resulted in haploid inducer lines with a maternal haploid induction phenotype (Zhu et al. 2020). Editing other genes, such as *CENTROMERE-SPECIFIC HISTONE 3* (*CENH3*) (Kuppu et al. 2020) or DUF679 domain membrane protein (*DMP*) using CRISPR/Cas9 could also significantly increase the haploid induction rate (HIR) (Zhong et al. 2019). These studies have greatly accelerated the application of this double haploid technology in plant breeding and hold great potential to accelerate breeding in long lifespan fruit crops.

### Employing woodland strawberry as a model for non-climacteric fruits

Fruit crops include climacteric fruits and non-climacteric fruits. Tomato is a canonical climacteric fruit, whereas the model plant for non-climacteric fruits is still poorly defined. As a versatile experimental plant system, the woodland strawberry (*Fragaria vesca*) has many advantages, including its short life cycle (3–6 months), ease of growth, and specific receptacle-derived accessory fruit (Shulaev et al. 2011). These advantages offer a good model to investigate the interaction between seeds and fruits during the fruit initiation and ripening process (Hollender et al. 2012; Kang et al. 2013a; Cappelletti et al. 2015; Härtl et al. 2017). More importantly, with the high throughput genome annotated, the feasible transformation system, and the high-efficient CRISPR/Cas genome editing system established for *Fragaria vesca*, it holds a great potential to serve as a model for functional studies in non-climacteric fruits. Hitherto, the CRISPR/Cas-based functional studies in the strawberry system have made great progress in illustrating the molecular basis of plant morphologic changes, fruit initiation, fruit ripening, and stress responses in strawberries (Feng et al. 2021a; Zhou et al. 2021; Guo et al. 2022; Mao et al. 2022). More and more cutting-edge technologies should be developed and applied to the strawberry system.

### Accelerating fruit crops breeding by genome editing systems

The traditional fruit crops breeding usually takes very long time and is labor-intensive because of the long life-cycle. Besides, most fruit crops genome have high levels of heterozygosity, it usually requires decades to produce homozygous plants with desired traits. In contrast, genome editing-based breeding is undoubtedly time-saving and easy to manipulate, simply by targeting and editing the candidate genes of desired traits. As the multi genes editing could be achieved simultaneously, a new conception called de novo domestication aroused more and more supports, in which a series numbers of desirable traits could be combined by targeting their responsive genes simultaneously through multiple genome-editing tools. This new technology could dramatically accelerate crop domestication from wild progenitors to cultivators and have been successfully applied in tomato (Li et al. 2018d; Zsogon et al. 2018). It also holds great potential in accelerating breeding in other fruit crops.

Forward genetic screening for the characterization of mutation with beneficial traits mostly relies on chemical, physical or transposon mutagenesis. However, the efficiency is usually low. CRISPR/Cas genome editing is also promising tools in genome wide screening. For this system, Cas9 is expressed along with a library sgRNA targeting many or all genes in plants. After transformation, regeneration, screening for desired trait, and sequencing of the sgRNA, final mutants were identified (Gaillochet et al. 2021). Large CRISPR libraries has become an advanced option for forward genetic screens. It could introduce mutation in genome-scale with high precision (Smith et al. 2017). CRISPR-dependent base editing screening is capable of identifying functional elements at single-base resolution (Cuella-Martin et al. 2021; Hanna et al. 2021). Besides, CRISPR screening can be combined with single-cell RNA-sequencing, providing new ways of deciphering gene function and genetic interaction (Doench 2018; Ford et al. 2019; Jin et al. 2020b; Replogle et al. 2020). It has been demonstrated that genome-wide mutant libraries has been developed in rice (Lu et al. 2017; Meng et al. 2017), tomato (Jacobs et al. 2017), maize (Liu et al. 2020a), and soybean (Bai et al. 2020; Gaillochet et al. 2021). However, compared with animal cells, the application of high-throughput screening in plants is still in its infancy. We anticipate that CRISPR mutant library will facilitate plant research on functional genomics study and significantly accelerate fruit crops breeding.

## Data Availability

Not applicable.

## Abbreviations

ABA: Abscisic acid
ABEs: Adenine base editors
ACC: 1-aminocyclopropane-1-carboxylic acid
*AGL6*: *AGAMOUS LIKE 6*
*AGL62*: *AGAMOUS-LIKE62*
*AGO7*: *ARGONAUTE7*
*ALS*: *ACETOLACTATE SYNTHASE*
*ARF4*: *AUXIN RESPONSE FACTOR 4*
AsCas12a: *Acidaminococcus* sp. BV3L6 Cas12a
*BBM*: *BABY BOOM*
*BFT*: *BROTHER OF FT AND TFL1*
bHLH: Basic Helix-Loop-Helix
BG: ABA hydrolyzation β-glucosidase
*BOP*: *BLADE-ON-PETIOLE*
BXW: Banana Xanthomonas Wilt
CBE: Cytosine base editors
*CBF1*: *C-REPEAT BINDING FACTORS 1*
*CCD8*: *CAROTENOID CLEAVAGE DIOXYGENASE 8*
*CEN4*: *CENTRORADIALIS 4*
CGBEs: C to G base edits
*CHS*: *CHALCONE SYNTHASE*
CmYLCV: *Cestrum yellow leaf curling virus*
*CNGCs*: *Cyclic nucleotide-gated ion channels*
*CNR*: *COLORLESS NON-RIPENING*
*CRC*: *CRABS CLAW*
CRISPR: Clustered regularly interspersed short palindromic repeats
CRISPRa: CRISPR-mediated gene activation
CRISPRi: CRISPR-mediated gene interference
CRISPR/Cas: Clustered Regularly Interspersed Short Palindromic Repeats and CRISPR-Associated Protein system
crRNA: CRISPR RNA
CsUbi: *Citrus sinensis* ubiquitin
Csy4: Csy-type (CRISPR system yersinia) ribonuclease 4
dCas: Catalytically inactivated Cas variants
dCas9: Catalytically inactivated Cas9
DDM1a: DECREASE IN DNA METHYLATION 1a
DH: Doubled haploid
DIPM: DspA/E-INTERACTING PROTEINS OF *Malus domestica*
*DMR6*: *DOWNY MILDEW RESISTANCE 6*
DMP: DUF679 domain membrane protein
*DOF9*: *DNA-BINDING WITH ONE ZINC FINGER 9*
DSBs: DNA double strand breaks
DsODN: Double-stranded oligodeoxynucleotide
*ENO*: *EXCESSIVE NUMBER OF FLORAL ORGANS*
*EPSPS*: *5-Enolpyruvylshikimate-3-phosphate synthase*
*ER*: *ERECTA*
eSpCas9: Enhanced specificity SpCas9
*ETR1*: *ETHYLENE RESPONSE 1*
*FLCL*: *FLOWERING LOCUS C-LIKE*
FM: Floral meristem
FnCas12a: *Francisella novicida* Cas12a
FON: *Fusarium oxysporum* f.sp.*niveum*
*FT*: *FLOWERING LOCUS T*
*FUL1*: *FRUITFULL*
GA: Gibberellic acid
GA20ox: GA 20-oxidases
GA3ox: GA3-oxidases
GABA: γ-Aminobutyric acid
GAD: Glutamate decarboxylase
GGP: GDP-L galactose phosphorylase
*GIFs*: *GRF-INTERACTING FACTORs*
*GRFs*: *GROWTH-REGULATING FACTORs*
GSC: Germ-line-specific Cas9 system
HDR: Homologous-directed repair
HI-Edint: Haploid induction editing
HIR: Haploid induction rate
*IAA9*: *AUXIN-INDUCED 9*
IMGE: Haploid inducer-mediated genome editing
*KIX*: *KINASE-INDUCIBLE DOMAIN INTERACTING*
LAM: LOSS OF AXILLARY MERISTEMS
LbCas12a: *Lachnospiraceae bacterium ND2006* Cas12a
LBD: LATERAL ORGAN BOUNDARY DOMAIN
*LCYε*: *Lycopene epsilon-cyclase*
*LOB1*: *LATERAL ORGAN BOUNDARY 1*
MAP K3: MITOGEN-ACTIVATED PROTEIN KINASE3
*MKK9*: *MITOGEN ACTIVATED PROTEIN KINASE KINASE 9*
*MLO*: *MILDEW RESISTANCE LOCUS O*
MMR: Mismatch repair
*MTL*: *MATRILINEAL*
MYB10R2R3: MYB TRANSCRIPTION FACTOR 10
*NAM1*: *NO APICAL MERISTEM 1*
nCas9: Nicked Cas9
NHEJ: Non-homologous end joining
*NOR*: *NON-RIPENING*
*NPR3*: *NONEXPRESSOR OF PATHOGENESIS-RELATED 3*
PAMs: Protospacer adjacent motifs
*PAT1*: *PHYTOCHROME A SIGNAL TRANSDUCTION 1*
*PDS*: *PHYTOENE DESATURASE*
pegRNA: Prime editing guide RNA
PEBP: Phosphatidylethanolamine-binding protein
PEG: Polyethylene glycol
PM: Powdery mildew
Pol III: RNA polymerase III
Pol II: RNA polymerase II
*PPD*: *PEAPOD*
*PR-1*: PATHOGENESIS-RELATED PROTEIN-1
*PR4*: *PATHOGENESIS-RELATED 4*
*ProSys*: PROSYSTEMIN
*PSK1*: *PHYTOSULFOKINE1*
PTG: Polycistronic tRNA-sgRNA cassette
RAP: REDUCED ANTHOCYANINS IN PETIOLES
RdDM: RNA-directed DNA methylation
*RDR6*: RNA-DEPENDENT RNA POLYMERASE 6
*RIN*: *RIPENING INHIBITOR*
RNPs: Ribonucleoproteins
S-gene: Susceptible gene
sgRNA: Single-guide RNA
*SGR*: *STAY-GREEN*
*SGS3*: SUPPRESSOR OF GENE SILENCING 3
*SP*: *SELF PRUNING*
*SPL*: *SQUAMOSA PROMOTER-BINDING PROTEIN-LIKE*
SpCas9: *Streptococcus pyogenes* Cas9
SRM1-like: SALT-RELATED MYB1-like
ssDNA: Single-stranded DNA
SSNs: Sequence-specific nucleases
SyGl: SHY GIRL
TALENs: Transcription activator-like effector nucleases
*TFL1*: *TERMINAL FLOWER 1*
TM6: TOMATO MADS BOX GENE6
*TMF*: *TERMINATING FLOWER*
ToBRFV: Tomato brown rugose fruit virus.
*TOM1*: *TOBAMOVIRUS MULTIPLICATION1*
TRV: Tobacco rattle virus
twinPE: Twin prime editing
uORF: Upstream open reading frame
VPE5: VACULAR PROCESSING ENZYME 5
*WUS*: *WUSCHEL*
*Xcc*: *Xanthomonas citri* subsp. *citri*
YUC: YUCCA
ZFNs: Zinc-finger nucleases
